# Dynamic Pathophysiological Insight into the Brain by NIR‐II Imaging

**DOI:** 10.1002/advs.202416390

**Published:** 2025-03-05

**Authors:** Si Chen, Hao Chen, Xinxin Li, Shuqing He, Kangquan Shou, Kun Qian, Zhao Fang, Feng Gu, Baisong Chang, Zhen Cheng

**Affiliations:** ^1^ Department of Neurology Xiangya Hospital Central South University Xiangya Road 88 Changsha 410008 China; ^2^ Molecular Imaging Program at Stanford (MIPS) Bio‐X Program Department of Radiology Canary Center at Stanford for Cancer Early Detection Stanford University California 94305‐5344 USA; ^3^ State Key Laboratory of Drug Research Molecular Imaging Center Shanghai Institute of Materia Medica Chinese Academy of Sciences Shanghai 201203 China; ^4^ Department of Neurology & Neurological Sciences Stanford University School of Medicine California 94305‐5122 USA; ^5^ State Key Laboratory of Advanced Technology for Materials Synthesis and Processing Wuhan University of Technology Wuhan 430070 China; ^6^ Bohai Rim Advanced Research Institute for Drug Discovery Yantai 264000 China

**Keywords:** BBB disruption, cerebral collateral circulation, crystalline organic nanoprobe, live‐brain imaging, NIR‐II fluorescence

## Abstract

Cerebral collateral circulation and blood‐brain barrier (BBB) are critically required to maintain the normal brain functions, a fact stressing the need for accurate and in vivo diagnostic tools that can afford valuable pathophysiological insight into the functioning of neurovascular unit in space and time. Currently, understanding of collateral perfusion and BBB evolution under both physiological and pathological conditions remains sparse, largely owing to limitations in methods for recording diminutive route of cerebral blood flow. Here, it is reported that highly crystalline semiconducting organic nanoprobes (named 4T‐BSA) composed of small‐molecule dye and bovine serum albumin showed vast potential for live‐brain vascular imaging in the second near‐infrared window (NIR‐II, 1000–1700 nm). The 4T‐BSA nanoprobes had superior imaging penetration depth in intact mouse brain with high signal‐to‐background ratio (SBR) of 6.0 and down to sub‐50‐µm spatial resolution of cerebral vasculature in three typical models of neurological pathophysiology. By visualizing the vascular collateral perfusion and albumin leakage, 4T‐BSA nanoprobes identified the pathological activities of brain associated with the arterial/venous collateral flow network and BBB disruption. It is anticipated that NIR‐II imaging of cerebral collateral circulation and BBB disruption will bring broad opportunities to address major medical challenges across timely, protective, and restorative interventions for neurological diseases.

## Graphical abstract

The functional integration of benefits from small semiconducting dyes and biocompatible serum proteins ushered in a new frontier of deep wide‐field in vivo imaging technique, affording valuable pathophysiological insight into the functioning of neurovascular unit in space and time.

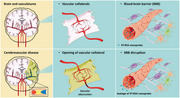

## 1 Introduction

The cerebral collateral circulation and blood‐brain barrier (BBB), two physiological hallmarks of cerebrovascular functions, play a vital role in supporting the neuronal activities of the brain.^[^ ^1^ ^]^ These two hallmarks integrate, coordinate, and respond to diverse physiological and pathological stimuli within the neurovascular unit that can ensure the central nervous system functioning such as BBB permeability regulation, angiogenesis, and collateral hemodynamic responses.^[^ ^2^ ^]^ Emerging advances clearly indicate that cerebral collateral perfusion is inextricably linked to the severity of cerebrovascular lesions,^[^ ^3^ ^]^ and BBB disruption can be regarded as an early detection biomarker for different neurological disorders.^[^ ^4^ ^]^ In this context, the in vivo monitoring of detailed dynamics of cerebral vasculatures and the evolution of pathological processes, especially the early‐phase BBB leakage, holds vast potential to clarify the working mechanisms of neurovascular unit under both neurophysiological and neuropathological conditions.

How to non‐invasively, dynamically detect the cerebral microvasculatures and BBB disruption processes has long fascinated neuroscientists.^[^ ^5^ ^]^ Currently, the in vivo longitudinal optical imaging, in principle, affords high spatial resolution maps of various hemodynamic parameters.^[^ ^6^ ^]^ However, quantification of blood perfusion by optical imaging technique is still in its infancy, not to mention the real‐time, wide‐field, and high‐resolution imaging of cerebrovascular systems acquired under deep‐brain penetration. Further developments of optical imaging are impeded by the absence of suitable probes with satisfied imaging capability, which would be characterized via high temporal and spatial resolution through intact scalp and skull. Furthermore, such probes should be able to pass the BBB and enter into brain parenchymal when the BBB is impaired.^[^ ^7^ ^]^

The emerging functional nanoprobes that can produce fluorescent signals in the second near‐infrared window (NIR‐II, 1000–1700 nm) have boosted the substantial development of the in vivo imaging technique.^[^ ^8^ ^]^ NIR‐II imaging mainly benefits from excellent signal‐to‐background ratios (SBR) inherent to low photon scattering and near‐zero tissue autofluorescence, enabling the NIR‐II probes competent for deep visualization of anatomical structure. Moreover, NIR‐II probes show salient imaging advantages as exemplified by sub‐10‐micron spatial resolution and high temporal resolution up to ≈20 frames per second.^[^ ^9^ ^]^ All these superior merits of NIR‐II probes, working together, effectively resolve the dynamics of blood flow on large field‐of‐view images in the range from micron to the entire mouse‐body.

Nowadays, the majority of the mainstream NIR‐II probes are composed of inorganic materials including single‐walled carbon nanotubes, rare‐earth nanocrystals, and semiconducting quantum dots.^[^ ^10^ ^]^ Notably, the long‐term in vivo retention and potential toxicity of inorganic materials will hinder their further bioimaging applications.^[^ ^11^ ^]^ The design of NIR‐II organic nanoprobes, similar in the structures and pharmacokinetics to FDA‐approved NIR‐I (700‐950 nm) fluorophores,^[^ ^12^ ^]^ would be regarded as a promising alternative to inorganic imaging agents. Very recently, our group had prepared a biocompatible small‐molecule organic probe (CH1055) with excellent renal clearance ability for NIR‐II in vivo imaging,^[^ ^13^ ^]^ but its low fluorescent quantum yield (**≈**0.3%) limited the deep‐tissue imaging. Despite the emerging progresses of design concepts that have been successful in the fabrication of organic NIR‐II probes, a bright fluorescence is generally achieved at the cost of complicated synthesis chemistries, e.g., molecular engineering method.^[^ ^14^ ^]^ In order to achieve live‐brain imaging through intact scalp and skull, challenges remain in the stability, brightness, toxicity and delivery of organic NIR‐II probes.

**Figure 1:**
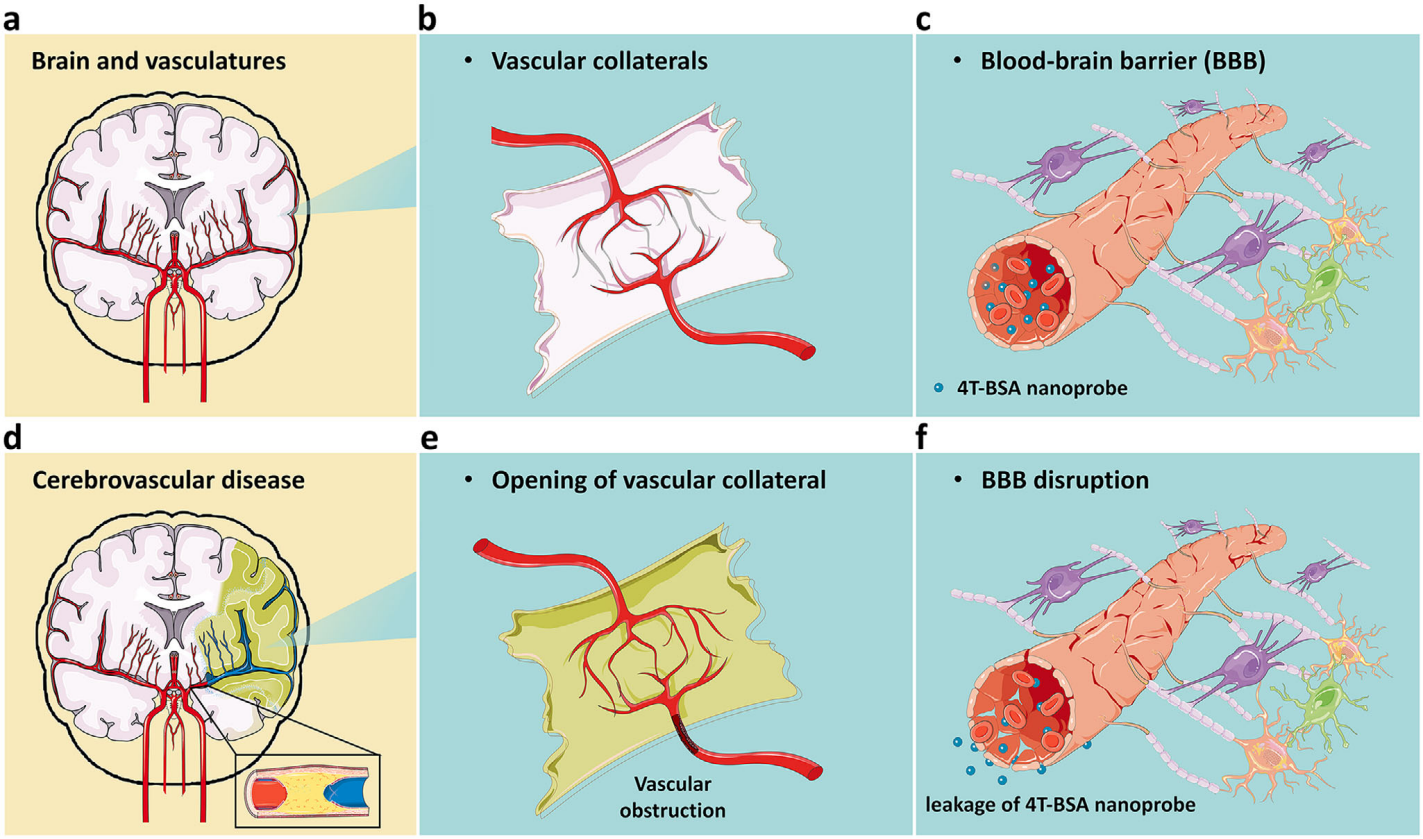
Schematic illustration of deep‐brain imaging of physiological and pathological processes in the second near‐infrared window. a–c) Physiological insights into the mouse brain (top panel). a) Scheme of brain and vasculatures. b) The cerebral collateral circulation, a network of tiny blood vessels, is naturally occurring artery‐to‐artery or arteriole‐to‐arteriole anastomoses and not open under physiological condition. c) Several tightly packed cell types such as pericytes and astrocytes contribute to the structure and function of blood‐brain barrier (BBB), which can impede influx of most compounds including 4T‐BSA nanoprobes from blood to brain. d–f) Pathological insights into the mouse brain (bottom panel). d) Scheme of cerebrovascular diseases as exemplified by middle cerebral artery occlusion (MCAO). e) Opening of vascular collateral blood flow in the neurological pathophysiology that can maintain residual blood flow to brain regions distal to arterial occlusion. f) 4T‐BSA nanoprobe can leak into brain parenchymal mainly owing to disruption of BBB integrity, one of the key factors in the pathological damage during cerebral ischemia.

Here, we developed a highly crystalline semiconducting organic NIR‐II nanoprobe (named 4T‐BSA), which was a novel type of molecular complexes via altering functional groups from CH1055 and then non‐covalently assembled with bovine serum albumin (BSA). In vitro and in vivo imaging was carefully performed in a narrow 1350 nm (NIR‐IIa) region compared with the common optical and magnetic resonance imaging of mouse models with typical neurological pathophysiology, for example middle cerebral artery occlusion (MCAO), cerebral venous sinus thrombosis (CVST), and pentylenetetrazol‐kindled epilepsy. Multiple lines of evidence indicated that 4T‐BSA nanoprobes showed high‐fidelity hemodynamic recording of deep cerebral vasculature and arterial or venous collateral responses under physiological condition (**Figure** **1**a–c). Importantly, due to high affinity for serum albumin, 4T‐BSA nanoprobes can accurately, non‐invasively and real‐time track subtle BBB permeability under pathological condition, especially the early‐phase BBB leakage (Figure 1d,e).

### 1.1 Synthesis and Optical Characterization of 4T‐BSA Nanoprobe

**Figure 2:**
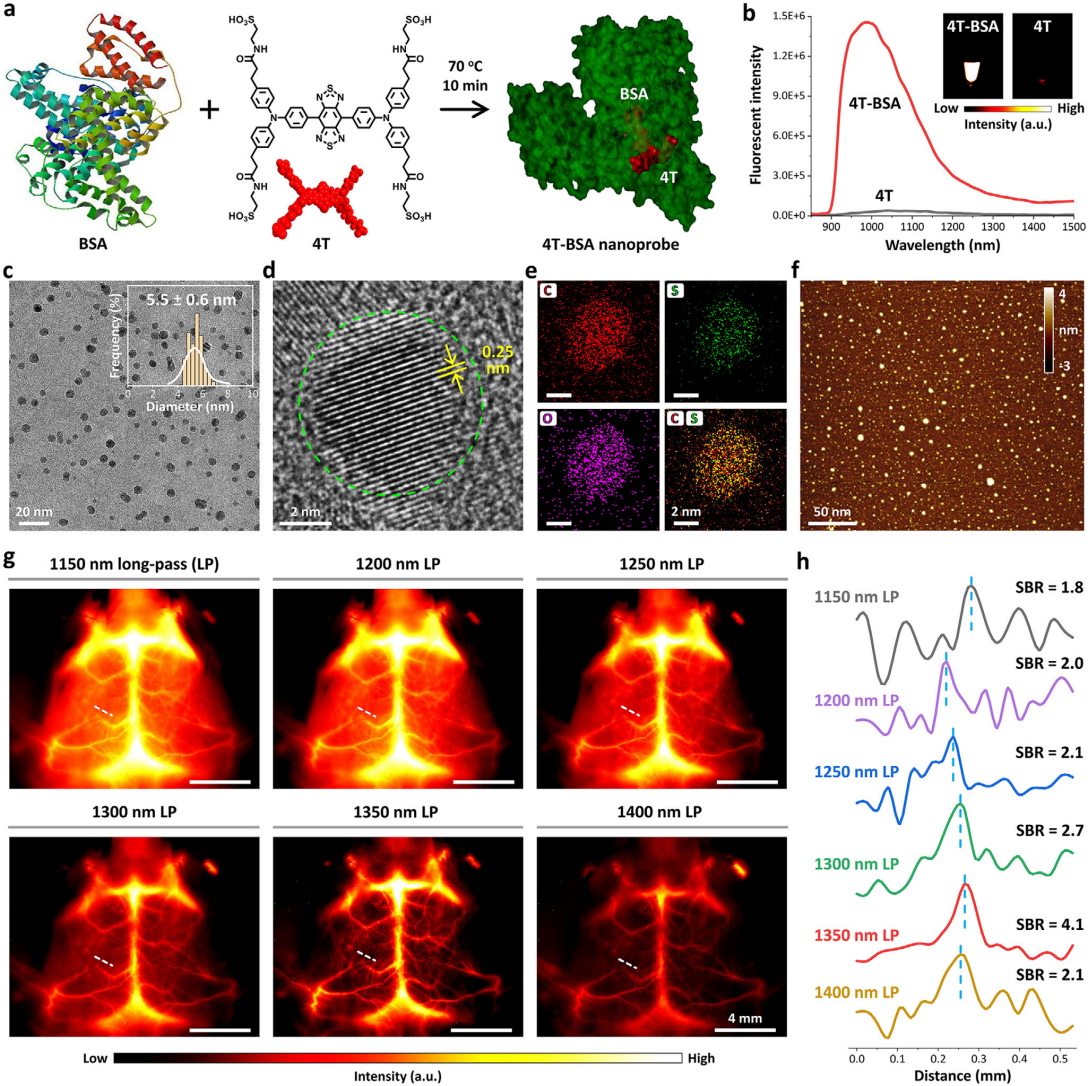
Structural and optical characterization of 4T‐BSA nanoprobe. a) Scheme of self‐assembly of 4T and BSA into highly crystalline semiconducting organic 4T‐BSA nanoprobes. b) Fluorescence emission spectra and NIR‐images (inset, 2000 ms exposure time, 1000 nm long‐pass filter) of 4T‐BSA nanoprobes and 4T under 808 nm excitation, respectively. c) Low‐magnification TEM image, d) HR‐TEM image, e) energy‐dispersive X‐ray spectroscopy elemental mapping, and f) AFM image of 4T‐BSA nanoprobe, respectively. Inset in (c) showed the corresponding size distribution histogram of 4T‐BSA nanoprobes by measuring over 100 particles. The distributions of C, S, and O elements were represented by red, green, and magenta color, respectively. g) Comparison of NIR‐II imaging of cerebral vasculatures under different long‐pass filters (1150–1400 nm). h) The cross‐sectional intensity profiles of cerebral vasculatures along the white‐dashed lines shown in g) NIR‐II images.

All the four carboxylic acid groups of CH1055 were replaced by sulfonic acid groups with more negative charge (named 4T), followed by assembled with BSA to achieve 4T‐BSA nanoprobes (**Figure** **2**a). Ultraviolet‐visible (UV–vis) spectra of 4T and 4T‐BSA nanoprobe clearly exhibited similar absorbance signals in the range of 320–1000 nm, while there was a strong absorbance peak at ≈280 nm for 4T‐BSA nanoprobe ascribing to the characteristic UV absorption peak of BSA molecule (Figure S1, Supporting Information). As shown in Figure 2b, the emission spectrum of 4T was almost a flat line parallel to abscissa. Very impressively, the self‐assembly of 4T and BSA resulted in a significant increase of fluorescence intensity under NIR‐II imaging system (inset of Figure 2b). The 4T‐BSA nanoprobe had a maximum fluorescence emission peak at **≈**990 nm, reaching up to over 110‐fold of emission intensity compared with 4T under 808 nm excitation. The quantum yield of 4T‐BSA was calculated to be 5.3% at 990 nm under 808 nm excitation (HiPCO SWCNTs as a reference 0.4%; Figure S2, Supporting Information).

The intriguing property of BSA‐activated fluorescent emission encouraged us to look into the microstructure of 4T‐BSA nanoprobe. The transmission electron microscopy (TEM) image and the corresponding size distribution histogram (Figure 2c) obviously demonstrated that colloidal stability of 4T‐BSA nanoprobes were spherical in shape with an average diameter of 5.5 ± 0.6 nm, whereas either 4T or BSA lacked the well‐defined nanostructures and morphology, leaving polydisperse in nature (Figure S3, Supporting Information). Upon careful measurement by high‐resolution TEM (HR‐TEM), the well‐developed lattice fringe with interplanar spacing of **≈**0.25 nm can be directly observed in 4T‐BSA nanoprobes (Figure 2d). The mapping of element distribution indicated the presence of thick S‐rich inner yellow domain, partially suggesting that the small‐molecule 4T was self‐assembled into the interior of BSA. Atomic force microscopy (AFM) confirmed that 4T‐BSA nanoprobes exhibited superior monodispersity in water and the dominant diameter located in the range of 3–7 nm (Figure 2f), consistent with TEM analysis.

Prior to bioimaging, the blood circulation time of 4T‐BSA nanoprobes with good biocompatibility (Figures S4 and S5, Supporting Information) was a big concern, since the long blood circulation time possibly affords a long imaging window for continuous monitoring of vascular dysfunction and recovery processes.^[^ ^15^ ^]^ NIR‐IIa intensity analysis of the major artery in hind limb (femoral artery) indicated a long visualization of 4T‐BSA nanoprobes in the blood vessel after 120 h post‐injection (Movie S1, Supporting Information). The corresponding blood circulation half‐life time was calculated to be ≈181 min by a two‐compartment pharmacokinetic model (Figure S6, Supporting Information). Moreover, the pharmacokinetic analysis hinted that 4T‐BSA nanoprobe can be excreted through the biliary system and completed excretion from the mouse body after ≈5 days post‐injection in the manner of organic dyes and serum protein. In parallel experiments by selecting sequential long‐pass filters from 1150 to 1400 nm, cerebral vasculatures at a longer wavelength window up to 1350 nm were clearer than those at a shorter wavelength window, after which point the signals of deep vessels became attenuated (Figure 2g). Further quantified SBR measurement of the cross‐sectional intensity profiles of cerebral vasculatures revealed that the maximum resolution of NIR‐II imaging was found to be under 1350 nm long‐pass filter (NIR‐IIa window) at an excitation of 808 nm (Figure 2h). In the following sections, deep‐brain imaging was deliberately performed in a NIR‐IIa window unless otherwise noted.

### 1.2 High‐Resolution NIR‐IIa Imaging of Cerebral Vasculatures

**Figure 3:**
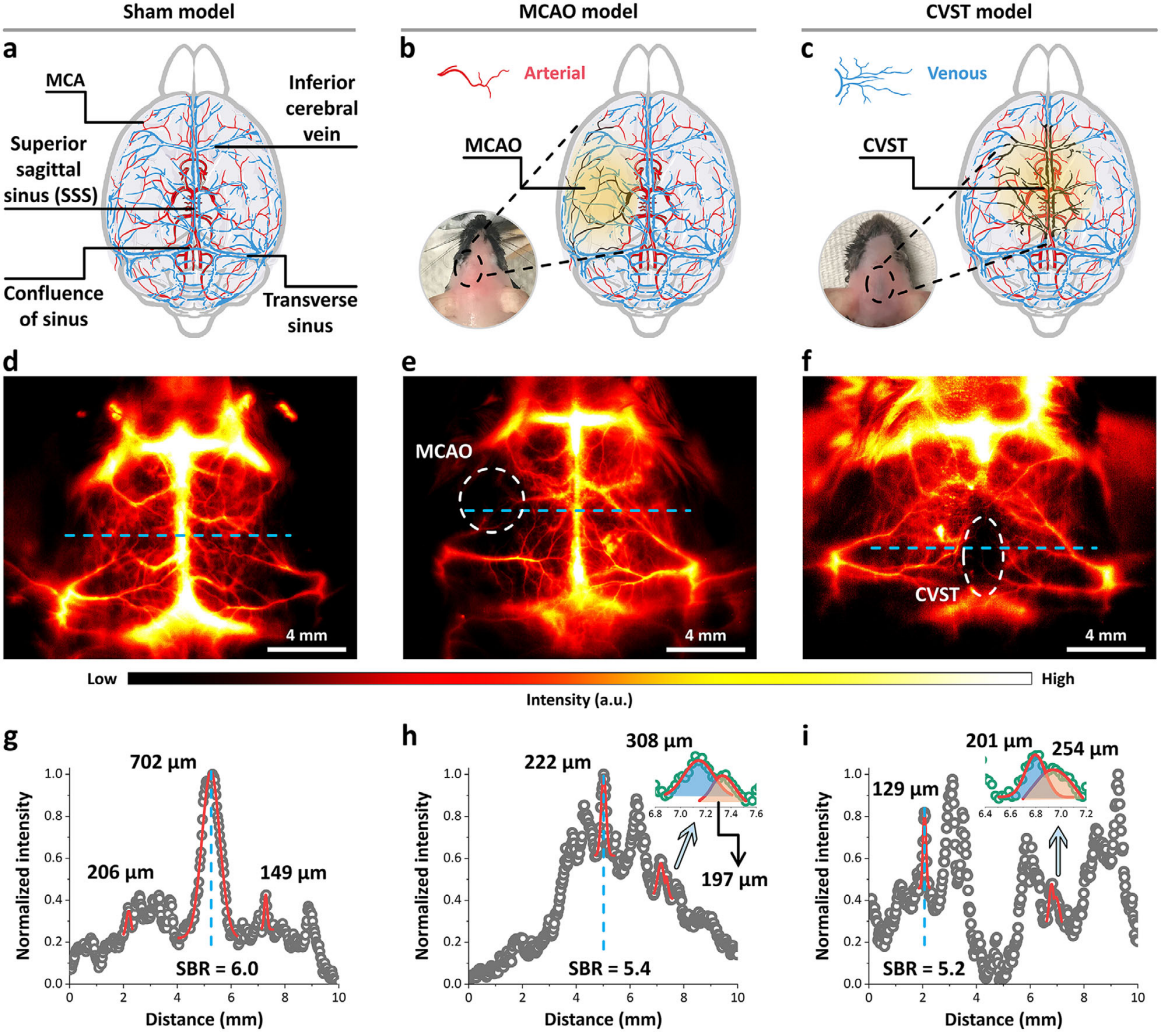
High‐resolution NIR‐IIa fluorescence in vivo imaging of cerebral vasculatures by 4T‐BSA nanoprobes in various neurologic pathophysiological mouse models. a–c) Schematic illustration of cerebral vasculatures of a) sham, b) MCAO, and c) CVST mouse models. The occluded vessels were marked with black color in the yellow domain. Both the sham and CVST mouse models were set up by removing hair. MCAO mouse models were set up with carotid surgery incision. d‐f) The typical NIR‐IIa live‐brain imaging of d) sham, e) MCAO, and f) CVST mouse models, respectively. The white‐dashed circle and ellipse represented the location of thrombosis in e) MCAO and f) CVST model, respectively. 4T‐BSA nanoprobe (35 nM) was injected intravenously into C57BL/6 mice with various neurologic pathophysiological mouse models followed by hair shaved, and then NIR‐IIa images of mouse cerebral vasculatures were obtained after injection (2000 ms exposure, color bar intensity ranging from 0 to 10 000). g–i) The cross‐sectional intensity profiles along the blue‐dashed lines outlined in (d–f) NIR‐IIa images, respectively. Quantitative studies of imaging quality were performed by fitting the recorded intensity profiles with the Gaussian function (red).

To investigate the feasibility of NIR‐II imaging to observe the pathophysiological processes of cerebral circulatory systems, 4T‐BSA and indocyanine green (ICG)‐BSA nanoprobes were injected intravenously with an equivalent dosage (35 nm) into three typical mouse models of neurological pathophysiology (n = 3 per group), respectively. Notably, ICG‐BSA was a NIR‐I nanoprobe with the maximum emission wavelength at **≈**810 nm (Figure S7, Supporting Information). The cerebrovascular systems of mouse brain were recorded on a large field‐of‐view image through intact scalp and skull within a narrow 1350–1650 nm imaging region under 808 nm laser illumination. In sharp contrast to the undistinguishable vasculatures and lower SBR calculated by ICG‐BSA nanoprobes in a NIR‐I (<900 nm) region (Figure S8, Supporting Information), cerebrovascular fluorescence imaging with 4T‐BSA nanoprobe in a NIR‐IIa window distinctly displayed much higher spatial resolution, irrespective of neurologic pathophysiological mouse models. In sham NIR‐IIa group (**Figure** **3**a,d), cerebral angiogram taken by 4T‐BSA nanoprobes perceptibly exhibited the middle cerebral artery (MCA), inferior cerebral vein, superior sagittal sinus (SSS), transverse sinus, confluence of sinus, together with plenty of cortical vessels and cerebral microvasculature in bilateral cerebral hemispheres under the scalp and skull. The SBR of SSS by 4T‐BSA nanoprobes in the NIR‐IIa region (Figure 3d) was calculated to be 6.0 ± 0.1, which was much higher than that of ICG‐BSA in a NIR‐I window (1.7 ± 0.2; *P* < 0.01, Figure S8, Supporting Information). The Gaussian‐fitted full width at half maximum (FWHM) of SSS imaged in a NIR‐IIa region was estimated to be 702 ± 3.9 µm (Figure 3g), whereas FWHM of SSS imaged by ICG‐BSA nanoprobe markedly increased to 1105 ± 8.2 µm (Figure S8, Supporting Information). Moreover, the FWHM of the chosen vasculatures of the left and right MCA territory was 206 ± 2.3 and 149 ± 4.1 µm, respectively. The sharp and exquisite NIR‐IIa imaging properties of 4T‐BSA nanoprobe potentially pave the way for addressing the imaging challenge of how to accurately, non‐invasively and real‐time recording of diminutive routes of cerebral blood flow.

The demonstration of above high‐resolution NIR‐IIa imaging of cerebral vasculature provided new opportunity to better understand vascular dysfunction and recovery processes. In the MCAO NIR‐IIa group, NIR‐II fluorescence signals of 4T‐BSA nanoprobes can be obviously observed in the right MCA territory in the contralateral hemisphere (Figure 3b,e), whereas little signal was found in the left (ipsilateral) hemisphere (marked with white‐dashed circle). The FWHM of cross‐sectional intensity profile of the selected vessels in the right MCA territory was determined to be 197 ± 7.1 µm and unachievable on the mirror artery in the left (Figure 3h), indicating the hypoperfusion within the MCA territory. Likewise, in the CVST NIR‐IIa group, 4T‐BSA nanoprobe can illuminate bilateral MCA territory (Figure 3f). The FWHM of selected vessels on the left MCA territory was 129 ± 5.2 µm with a high SBR of 5.2 ± 0.1 (Figure 3i), while little fluorescence signals were monitored in SSS region. Therefore, NIR‐IIa imaging enabled accurate location of thrombosis in the CVST model.

### 1.3 Quantitative Analysis of Hemodynamic Cerebral Perfusion

**Figure 4:**
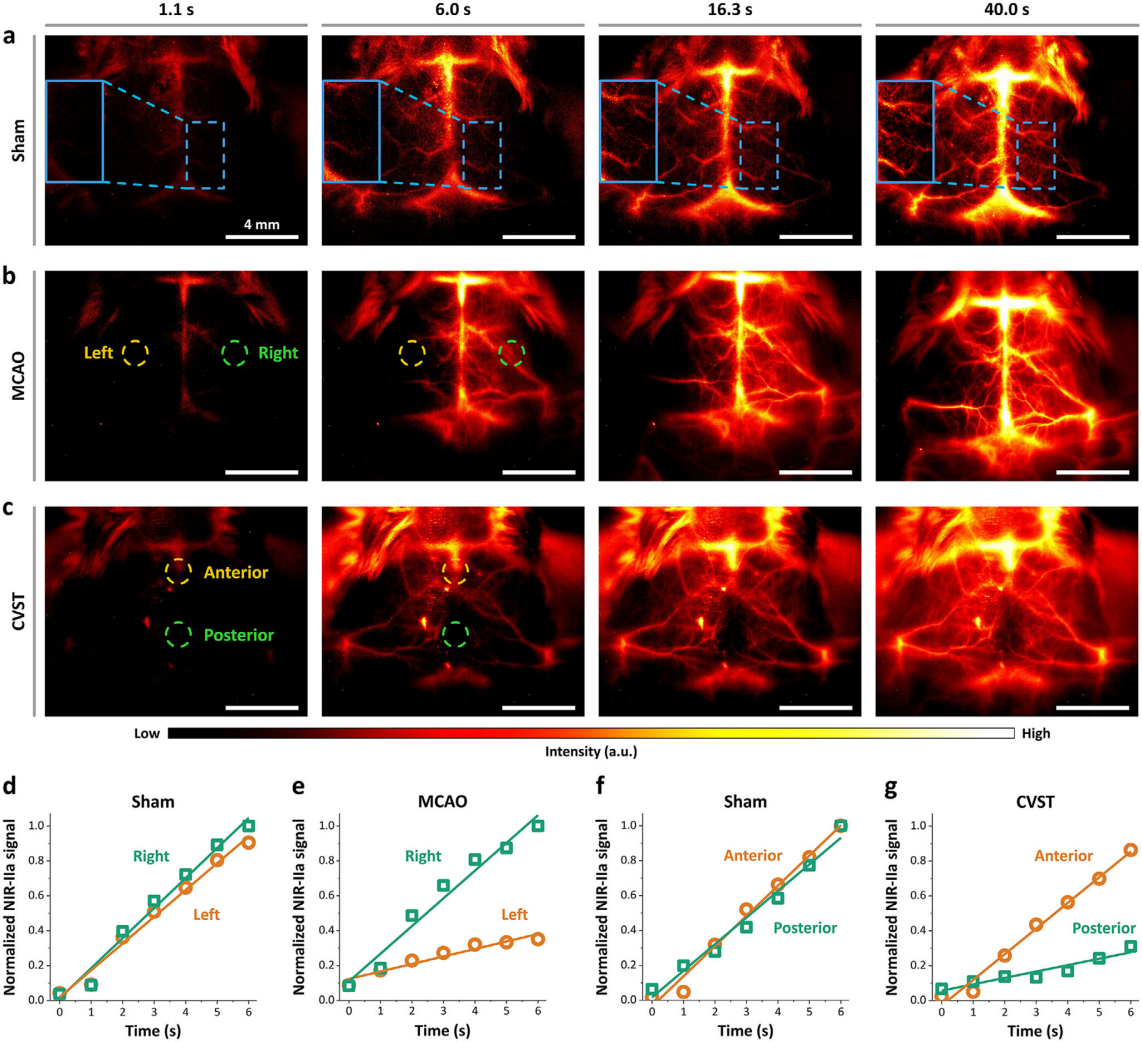
NIR‐IIa in vivo imaging by 4T‐BSA nanoprobes for quantitative analysis of hemodynamic cerebral perfusion in various neurologic pathophysiological mouse models. a) Time‐course NIR‐IIa frames in sham group (1000 ms exposure, color bar ranges from 0 to 10 000) at indicated time points of 1.1, 6.0, 16.3, and 40 s post‐injection of 4T‐BSA nanoprobes (35 nm) without craniotomy. Time‐course NIR‐IIa frames in b) MCAO and c) CVST mouse models at indicated time points by intravenous injection of 4T‐BSA nanoprobes. Normalized NIR‐IIa fluorescence intensity of the bilateral MCA territory versus time in d) sham and e) MCAO models. Normalized NIR‐IIa fluorescence intensity of anterior and posterior region of SSS territory versus time in f) sham and g) CVST models. The yellow and green‐dashed circles in panel (b,c) represented the region of interest for perfusion analysis in different ischemic models.

To study the regional cerebral blood flow, a valid measure of local neuronal activity,^[^ ^16^ ^]^ 4T‐BSA nanoprobe (35 nM) was administered into C57BL/6 mouse of sham, MCAO and CVST models with the tail vein injection, respectively. Time‐course NIR‐IIa frames (1350 nm long‐pass filter, 808 nm excitation and imaging rate of 1 frame per second) were recorded to investigate signal fluctuation across the field of view of the brain without craniotomy. In sham NIR‐IIa group, NIR‐II fluorescent intensity rapidly increased with an extension of post‐injection time (**Figure** **4**a). As expected, NIR‐IIa signal intensity of the left MCA territory nearly equaled the corresponding right one, inferring an identical perfusion rate of bilateral MCA territory (Figure 4d).

In the MCAO group, the fluorescence signal of 4T‐BSA nanoprobes (outlined by green‐dashed circle in Figure 4b) showed up immediately in the right MCA (contralateral) territory within 1 s post‐injection. Notably, fluorescence signals in the right MCA rapidly increased and plateaued at ≈40 s post‐injection, whereas little NIR‐IIa signal appeared in the left MCA (ipsilateral) territory as shown by yellow‐dashed circle (Movie S2, Supporting Information). Analysis of dynamic fluorescence signal fluctuations indicated much‐reduced blood perfusion in the left MCA region of the brain (Figure 4e). For example, the cerebral blood perfusion rate of the right MCA territory reached up to about fivefold higher than that of the corresponding left ischemic MCA territory.

Moreover, there was no clear difference of NIR‐IIa signals between the posterior and anterior regions of SSS in the sham group (Figure 4a,f). Very impressively, NIR‐IIa fluorescence signals can be observed instantly in the anterior region of SSS (yellow‐dashed circle in Figure 4c) after injection of 4T‐BSA nanoprobes in the CVST group (Movie S3, Supporting Information). Meanwhile, NIR‐IIa signals were almost negligible in the posterior region of SSS (green‐dashed circle in Figure 4c), which represented the sites of thrombosis. As summarized in Figure 4g, the cerebral blood perfusion rate in the anterior region of SSS was approximately fourfold higher than in the posterior region, suggesting the venous reflux disorder due to the sinus thrombosis (occlusion) in the posterior region of SSS.

### 1.4 Dynamic Real‐Time Recording of Collateral Circulation

During the ischemic period, collateral perfusion networks with branches of the major cerebral arteries can ensure the supply of blood flow to injured brain tissues.^[^ ^17^ ^]^ Notably, no ideal or specific imaging modality, so far, is available for dynamic demonstration of cerebral microcirculation and opening of secondary collateral perfusion in vivo. The potential of 4T‐BSA nanoprobe for accurate measurement of the cerebral microvascular arterial and venous collaterals was conducted in the event of the proximal occlusion of large intracranial blood vessels (MCA or SSS). The entire mouse head was mounted on an imaging stage beneath the laser and then recorded continuously during a 90 min period after intravenous injection of 4T‐BSA nanoprobe (35 nm) into C57BL/6 mouse of MCAO and CVST models, respectively.

**Figure 5:**
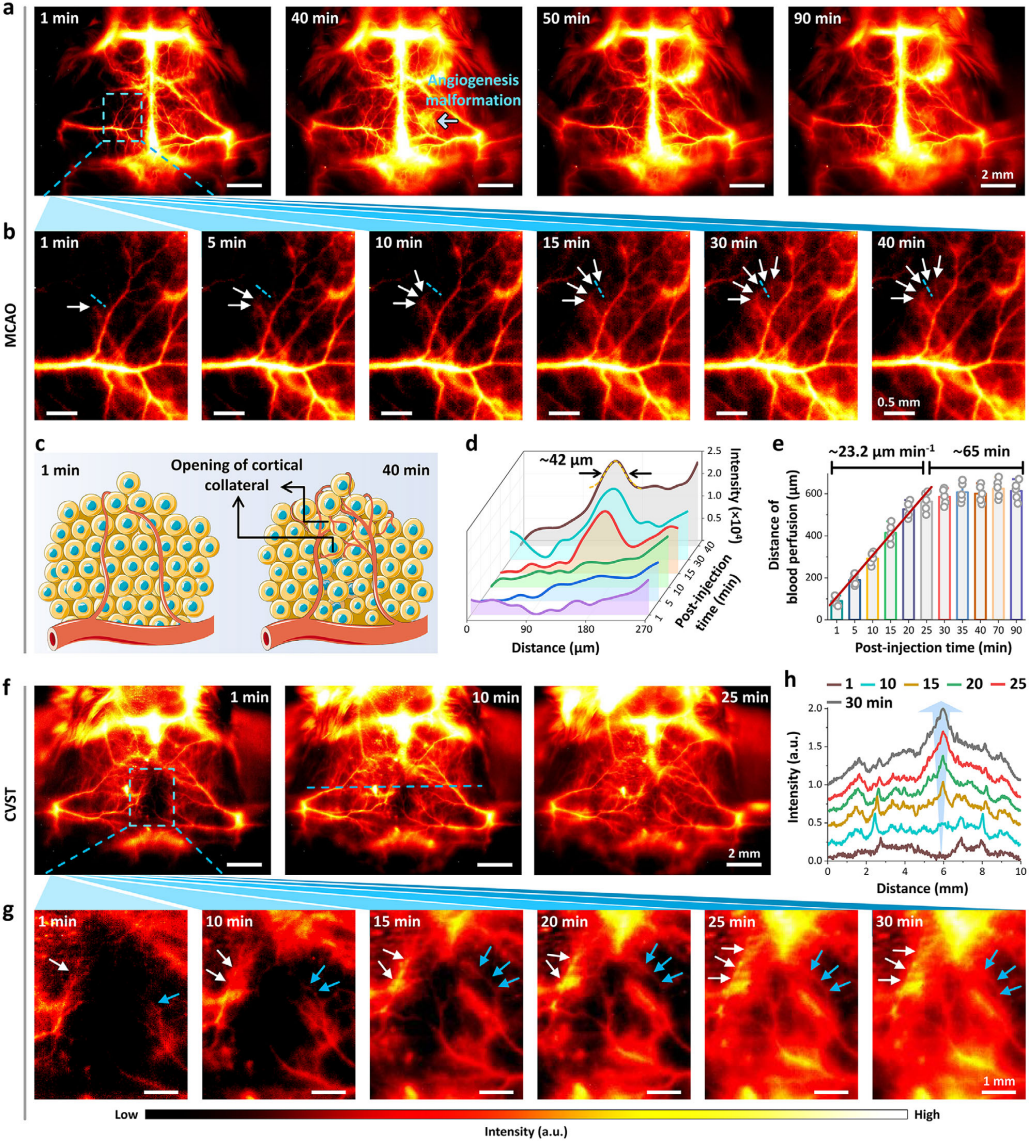
In vivo real‐time recording of the evolution of arterial or venous collateral flow network in the brain. a) Time‐course NIR‐IIa imaging (2000 ms exposure time) of cerebral vasculatures in the C57BL/6 mouse MCAO model. The entire mouse head was mounted on an imaging stage and detected continuously after an intravenous injection of 4T‐BSA nanoprobes (35 nm). Blue‐dashed rectangle and arrow in a) (1, 40 min) showed a part of occluded MCA territory and an angiogenesis malformation, respectively. b) Time‐course NIR‐IIa frames in a by high magnification (10×) within 40 min post‐injection of 4T‐BSA nanoprobe. Single arterial collateral was observed to be perfused (the opening of collateral vessels depicted by white arrows). c) Schematic illustration of evolution of collateral flow network. d) The cross‐sectional intensity profiles along the blue‐dashed lines in b) of enlarged NIR‐IIa images. Gaussian fit curve was shown by orange‐dashed line. e) The distance of blood perfusion (represented with the white arrows in b) against time over a course of 90 min post‐injection of 4T‐BSA nanoprobes. f) Time‐course NIR‐IIa imaging (2000 ms exposure time) of cerebral vasculatures in C57BL/6 mouse CVST model. Blue‐dashed rectangle in (f) (1 min) exhibited the area of occluded SSS. g) Time‐course NIR‐IIa frames in (f) under a high magnification (5×) during 30 min post‐injection of 4T‐BSA nanoprobes. Both white and blue arrows displayed the perfusion of venous collaterals. h) Series analysis of the cross‐sectional intensity profiles along blue‐dashed line in panel (f) against time over a course of 30 min post‐injection of 4T‐BSA nanoprobes. Data in (e) were mean ± s.d.

In the MCAO group, NIR‐IIa dynamic images with impressive clarity can be real‐time captured during a 90 min period under low magnification (2.5×) to observe the entire mouse head (**Figure** **5**a; Figure S9, Supporting Information). Of note, the blue arrow in NIR‐IIa dynamic image (40 min) represented an angiogenesis malformation, as evidenced by bright emission intensity of 4T‐BSA nanoprobe in brain vessel. On zooming into the left MCA territory, the region of interest (blue‐dashed rectangle in Figure 5a) was monitored under high magnification (10×) of NIR‐IIa dynamic imaging to clarify the details of blood hypoperfusion. As shown in the ischaemic region, the blood perfusion into a tiny single capillary at ≈15 min post‐injection of 4T‐BSA nanoprobe was unambiguously observed (white arrows in Figure 5b). The cross‐sectional intensity profiles across the blood perfusion regions by blue‐dashed lines in Figure 5b established the emergence of tiny blood vessel after **≈**15 min post‐injection of 4T‐BSA nanoprobes (Figure 5d). Such newly formed vessels were characterized by sharp peaks with FWHM of ≈42 µm. The NIR‐IIa imaging of blood perfusion suggested the opening of cortical arterial collaterals, which could support the brain tissue survival over the occluded left MCA territory (Figure 5c). Meanwhile, the flow of 4T‐BSA nanoprobe can be utilized to evaluate the blood perfusion dynamics associated with the arterial collateral circulation (Figure 5e). The distance of blood perfusion increased linearly at an early stage of post‐injection time (<25 min) of 4T‐BSA nanoprobe, and the blood perfusion rate can be estimated to be **≈**23.2 µm min^−1^. Particularly, at a late post‐injection time, the perfusion rate reached a plateau within 90 min measurement time window. The above blood perfusion study made on the basis of the evolution of arterial collateral flow network in the brain of MCAO model identified that the maximal opening of intrinsic arterial collaterals maintained at least 65 min (Figure 5e), enabling sufficient blood flow to support the brain viability during acute ischemic diseases.

In the CVST model, cerebral venous collateral perfusion can be facilely measured surrounding the site of venous sinus occlusion (Figure 5f). Taking a close look at the sequential magnified NIR‐IIa images within 30 min post‐injection of 4T‐BSA nanoprobes, numerous and complicated collateral anastomose development was clearly detected over the occluded venous sinus territory (Figure 5g). Moreover, multiple collateral connection was found to move from cerebral blood vessels around the thrombosis (medium‐posterior region of SSS) to the anterior region of SSS (Figure 5g, white and blue arrows). Taken together, NIR‐IIa imaging by 4T‐BSA nanoprobe can elucidate high‐resolution continuous real‐time monitoring of collateral blood perfusion. Quantitative analysis of the cross‐sectional intensity profiles along the same vessels and anastomose regions further demonstrated cerebral venous collateral perfusion around the occluded venous sinus territory (Figure 5h).

### 1.5 High‐Fidelity Dynamic Recording of BBB Permeability

**Figure 6:**
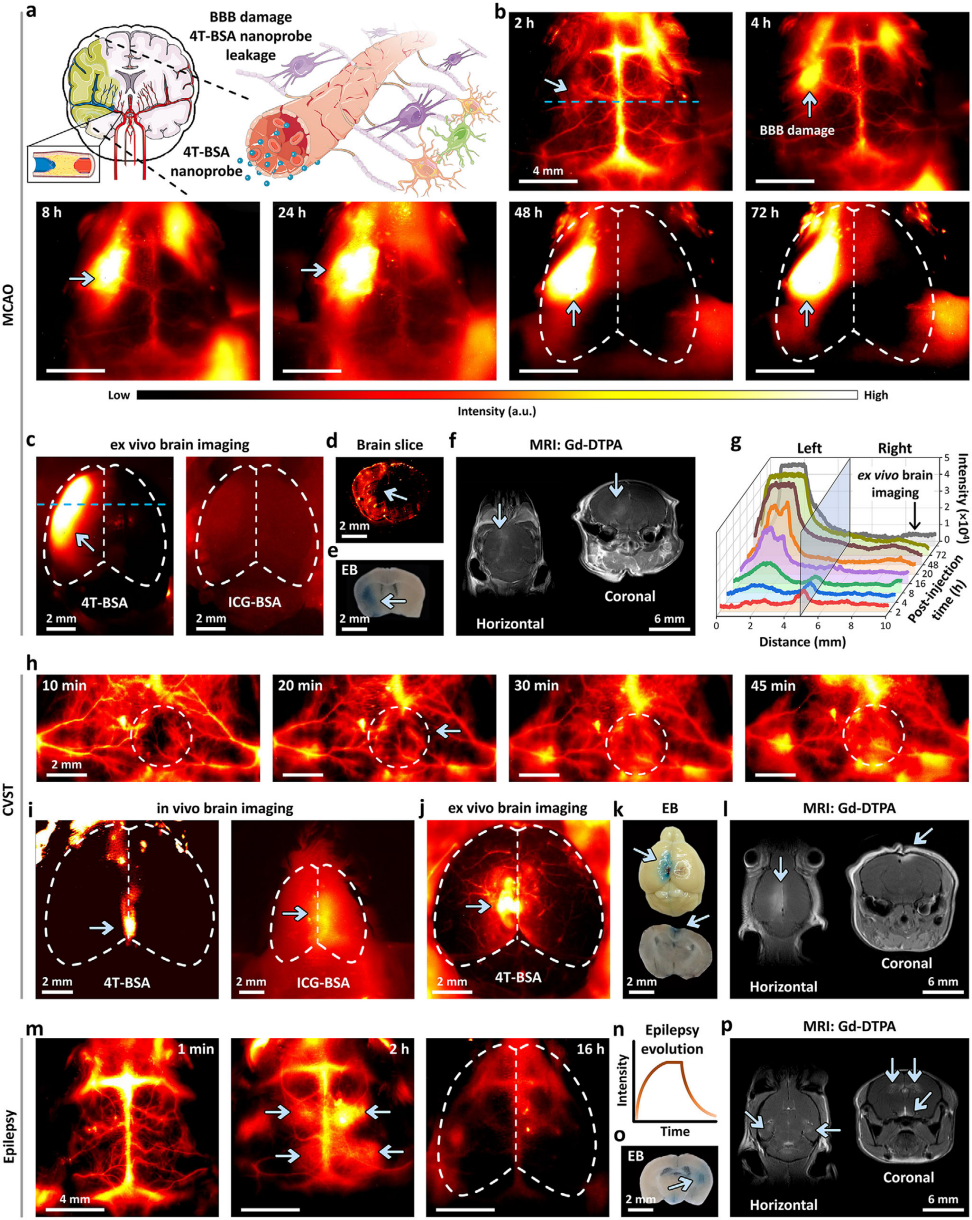
High‐fidelity dynamic monitoring of BBB permeability by NIR‐IIa imaging in MCAO, CVST, and epilepsy mouse models, respectively. a) Schematic illustration of 4T‐BSA nanoprobes for the live‐mouse cerebrovascular imaging and BBB leakage evaluation in MCAO model. b) Non‐invasive NIR‐IIa brain imaging from 2 to 72 h after reperfusion of the 4T‐BSA nanoprobe. c) Ex vivo NIR‐IIa imaging by 4T‐BSA nanoprobe (left) and NIR‐I imaging by ICG‐BSA (right) of entire mouse brain in MCAO model, respectively. d) Ex vivo NIR‐IIa imaging of coronal brain slice with a thickness of 20 µm at 72 h after reperfusion. e) Ex vivo imaging of coronal brain slice with a thickness of 1 mm in MCAO mouse at 72 h after reperfusion with intravenous injection of 1.25 µm Evans blue. f) Post‐contrast T_1_‐weighted MRI images in horizontal and coronal sections after Gd‐DTPA injection (0.1 mmol kg^−1^) into the mouse MCAO model at 72 h after reperfusion. g) The cross‐sectional intensity profiles along the blue‐dashed line in panel (b) at different post‐injection time points from 2 to 72 h. It was important to point out that the dark‐grey curve represented the cross‐sectional intensity profile along the blue‐dashed line shown in ex vivo NIR‐IIa imaging for brain in the left panel of (c). h) Dynamic in vivo NIR‐IIa images of 4T‐BSA nanoprobe permeability zoomed into the SSS regions of CVST model. The white‐dashed circle represented the site of the thrombosis. i) The in vivo NIR‐IIa imaging using 4T‐BSA (left) and NIR‐I imaging by ICG‐BSA nanoprobes (right) of the whole brain of CVST mouse model, respectively. j) Ex vivo NIR‐IIa imaging of mouse brain of CVST model at 16 h post‐injection of 4T‐BSA nanoprobes. k) Ex vivo imaging of brain (top panel) and a coronal brain slice (bottom panel, thickness of 1 mm) in CVST mouse at 16 h after reperfusion with intravenous injection of 1.25 µm Evans blue. l) Post‐contrast T_1_‐weighted MRI images (horizontal and coronal sections) after 0.1 mmol kg^−1^ Gd‐DTPA injection into CVST mouse model at 16 h after reperfusion. m) Non‐invasive NIR‐IIa brain imaging from 1 min to 16 h after an intravenous injection of 4T‐BSA nanoprobe into epilepsy mouse model. n) The epilepsy evolution by monitoring BBB permeability against reperfusion time over a course of 16 h. o) Ex vivo imaging of coronal brain slice (thickness of 1 mm) from the epilepsy mouse (2 h after seizure) with intravenous injection of 1.25 µM Evans blue. p) Post‐contrast T_1_‐weighted MRI images in horizontal and coronal sections after Gd‐DTPA injection (0.1 mmol kg^−1^) into epilepsy mouse model at 2 h after reperfusion. Notably, all the blue arrows showed the sites of BBB permeability in various mouse models. In each group, experiment performed with three independent mice with similar results.

Despite accumulating evidence for the role of BBB permeability changes in the pathogenesis of common neurological diseases, there is a lack of high‐fidelity approaches for investigating BBB properties in tiny cerebral blood vessels under both physiological and pathological conditions. To this end, we established three typical mouse models of neurological pathophysiology, e.g., MCAO, CVST, and epilepsy, which can be used to accurately mimic different BBB disruption behaviors (**Figure** **6**a). In addition to NIR‐IIa imaging by 4T‐BSA nanoprobes, other typical NIR‐I, magnetic resonance imaging (MRI), and albumin‐tagged Evans blue (EB) brain imaging modalities were also performed carefully for the characterization of relative changes of BBB properties.

In order to obtain dynamic in vivo NIR‐IIa brain imaging in the MCAO group, the whole mouse head was mounted on an imaging stage beneath the laser. Non‐invasive NIR‐IIa imaging was then carried out immediately after the injection of 4T‐BSA nanoprobes within a narrow 1350–1650 nm imaging region. The remarkable fluorescence signals (blue arrows in Figure 6b) were found in MCAO model mice from 2 to 72 h after reperfusion, demonstrating extravasation and accumulation of more 4T‐BSA nanoprobes over ischemic hemisphere compared with its contralateral side. Moreover, the time‐course signal analysis of ischemic MCAO territory revealed that the leakage signal intensity and area of 4T‐BSA nanoprobe increased and expanded slowly over time (Figure 6g; Figure S10, Supporting Information). It should be mentioned that the ex vivo brain NIR‐IIa imaging followed by removing the whole mouse brain from skull at 72 h after reperfusion can retain intense fluorescence signals over the ischemic MCA territory (grey curve in Figure 6g), demonstrating apparent extravasation of 4T‐BSA nanoprobes (Figure 6c, left). Meanwhile, the NIR‐IIa microscopic imaging of brain coronal slice with **≈**20 µm thickness confirmed the behavior of BBB disruption (Figure 6d) as evidenced by apparent extravasation of 4T‐BSA nanoprobe in ischemic hemisphere of MCAO territory. In the ex vivo EB group (Figure 6e), which is a standard ex vivo marker for the BBB leakage in brain slice, the EB extravasation was found to show a similar spatial distribution to 4T‐BSA nanoprobes over the ischemic MCAO territory in the MCAO model, but at the cost of ≈35.7‐fold higher in dosage (1.25 µm) and 50‐fold larger in slice thickness (1 mm) than 4T‐BSA nanoprobes (35 nm, 20 µm). In this regard, 4T‐BSA nanoprobe may be helpful to efficiently clarify the BBB evolution under pathological conditions.

To assess the extravasation of plasma albumin in the brain parenchyma, the accumulated 4T‐BSA nanoprobe signal over the ischemic MCAO territory was measured by time‐series analysis of cross‐sectional intensity profiles along the blue‐dashed lines in Figure 6b. The trapped NIR‐IIa signals will spread over as large a region as MCAO territory at 8 h post‐injection, and surpassed the core region of MCAO territory after 16 h (Figure 6g). This finding suggested that the edema formed region seemed to precede the domain of MCAO territory and was accompanied by the extravasation of plasma constituents (Figure 6c, left).

Moreover, for the CVST NIR‐IIa group, additional cerebral venous ischemia experiments were performed followed by monitoring time‐course NIR‐IIa frames from 1 to 45 min after intravenous injection of 4T‐BSA nanoprobes (35 nM). As shown in Figure 6h, dynamic in vivo NIR‐IIa images with a higher magnification (5×) hinted the leakage of 4T‐BSA nanoprobe at 10 min post‐injection. The accumulation of 4T‐BSA nanoprobes in brain parenchyma close to the superior sagittal sinus was traced successfully every 5 min within 45 min measurement time window. Of note, in vivo imaging of CVST mouse brain at 16 h post‐injection of 4T‐BSA nanoprobes revealed that NIR‐IIa signal, as well as the observed BBB disruption, was retained over the ischemic SSS territory (Figure 6i, left). In addition, ex vivo imaging of CVST mouse brain identified the extravasation of 4T‐BSA nanoprobes surrounding the SSS (Figure 6j), very similar to ex vivo imaging of brain from CVST mouse (16 h after reperfusion) with an intravenous injection of EB dye (Figure 6k).

To further study the sensitivity and penetration depth of 4T‐BSA nanoprobe for detecting BBB permeability, pentylenetetrazol‐kindled mouse model of epilepsy was set up by recording latency and intensity for 1 h after a single intraperitoneal injection of pentylenetetrazol, and then 4T‐BSA nanoprobes (35 nm) were injected intravenously to observe the BBB integrity. Under the NIR‐IIa imaging window, typical BBB disruption pattern can be found in the brain with pentylenetetrazol‐induced seizures, as evidenced by the extravasation of 4T‐BSA nanoprobes in multiple areas. For example, at 2 h post‐injection of 4T‐BSA nanoprobe, intense leakage was observed in the bilateral parietal‐temporal cortex and around hippocampus areas (Figure 6m). Interestingly, NIR‐IIa intensity ascribing to BBB leakage was almost negligible after 16 h post‐injection of 4T‐BSA nanoprobe (Figure 6m,n), hinting the reversible BBB disruption behavior for a single episode of epilepsy. The ex vivo experiment with EB dye established BBB leakage around the hippocampus and midbrain, while a faint blueish stained on parieto‐temporal cortex (Figure 6o). The above imaging data identified that 4T‐BSA nanoprobe allowed for non‐invasive in vivo monitoring of subtle BBB permeability with a high degree of clarity and deep penetration in intact mouse brains.

As a comparison, in the NIR‐I group, ICG‐BSA nanoprobes (35 nM) were injected intravenously into three typical models of neurological pathophysiology, respectively. Non‐significant variation was monitored in vivo around the ischemic territory of MCAO, CVST, and epilepsy mouse models (right panels of Figure 6c,i; Figure S11, Supporting Information). For example, ICG‐BSA nanoprobes with a short emission wavelength cannot achieve high‐quality ex vivo NIR‐I imaging of the brain removed from the skull at 72 h after reperfusion in MCAO mouse model (Figure 6c, right panel). For the MRI control group, post‐contrast T_1_‐weighted imaging was conducted immediately after Gd‐DTPA radiotracer injected intravenously ascribing to its very short plasma half‐life.^[^ ^18^ ^]^ It was noteworthy to point out that contrast‐enhancement imaging can only be detected around the margin of ischemic territory (Figure 6f,l,p) irrespective of MCAO, CVST, or epilepsy neurological mouse model. The union of above imaging modalities supported a convincing result that 4T‐BSA nanoprobes showed vast potential for high‐fidelity and persistent recording of BBB permeability in a NIR‐IIa imaging window.

## 2 Discussion

The cerebral collateral perfusion from vascular obstruction and leakage from early‐phase BBB disruption, a global pathophysiological activity within the neurovascular unit, offer a pathological basis for widespread neurological dysfunctions.^[^ ^19^ ^]^ Specifically, dynamical vascular processes alter cerebral microvessel perfusion, angiogenesis as well as BBB permeability which is represented as increased levels of serum albumin, fibrinogen and autoantibody.^[^ ^20^ ^]^ It will be important to develop molecular imaging method that non‐invasively and dynamically records specific microvasculature circulatory and BBB disruption processes. Vascular collateral circulation acts as a compensatory network to restore blood flow to ischemic regions.^[^ ^21^ ^]^ Adequate collateral flow reduces the severity and duration of hypoxia, thereby mitigating endothelial cell activation and preserving BBB integrity. Our findings revealed that highly crystalline organic 4T‐BSA nanoprobes achieved high‐fidelity hemodynamic visualization of deep cerebral vasculatures in typical models of neurological pathophysiology (i.e., MCAO, CVST, and epilepsy) benefiting from low photon scattering, minimal tissue absorption, and near‐zero background autofluorescence.

4T‐BSA nanoprobes resolved the dynamics of blood flow on a large field‐of‐view image of the whole mouse brain through intact scalp and skull. NIR‐IIa imaging with 4T‐BSA nanoprobes clearly demonstrated the successful set‐up of different neurologic pathophysiology models. The MCAO model can evaluate the ability of 4T‐BSA nanoprobes to penetrate dynamically compromised BBB and target ischemia‐driven biomarkers. The CVST model was used to emphasize the specificity of 4T‐BSA nanoprobes for thrombosis imaging in low‐flow venous systems, highlighting its potential for differentiating arterial versus venous thrombosis. The epilepsy model aimed to validate the sensitivity of 4T‐BSA nanoprobes to detect early‐stage microvascular abnormalities. 4T‐BSA nanoprobes can quantitatively study hemodynamic cerebral perfusion rate by analysis of dynamic fluorescence signal fluctuations. Furthermore, NIR‐IIa dynamic imaging accurately clarified the details of blood hypoperfusion and established the opening of either arterial or venous collaterals corresponding to the ischemic regions in MCAO or CVST models. The ability of 4T‐BSA nanoprobes to simultaneously map perfusion deficits (collateral failure) and hint at BBB leakage (via signal retention) may provide a dual readout for stratifying stroke severity. The 4T‐BSA signal in the anterior SSS highlighted the regions with residual collateral drainage, which can be targeted for thrombolytic therapy to restore venous outflow and alleviate BBB stress. These new collaterals or collateral anastomose networks might allow brain tissue survival over the occluded vessel territory.

Several pathologies that cause neural damages and degeneration always show an early phase involving BBB disorders. Various imaging techniques have been settled to monitor BBB disruption in injured animal and disease models, yet have extra limitations. EB is known to be a standard ex vivo marker for evaluating BBB permeability in brain slices of animal.^[^ ^22^ ^]^ However, requirement of sacrificing animals makes it unsuitable to real‐time track BBB process in vivo. Moreover, contrast‐enhanced MRI with Gd‐DTPA is of great value for non‐invasive investigating BBB disruption,^[^ ^23^ ^]^ but its clinical trial is impeded by inherently low sensitivity, restricted spatial and temporal resolution. In addition, resulting from the weak binding affinity to plasma albumin and low molecular weight of Gd‐DTPA (550 Da), it cannot differentiate various stages of BBB disruption. Compared with the spatial extravasation patterns associated with EB or Gd‐DTPA, 4T‐BSA nanoprobes enabled more persistent, rapid, and accurate detection of pathophysiological processes of subtle BBB disruption in vivo. Due to excellent chemical and optical properties of 4T‐BSA nanoprobes, BBB permeability can be clearly visualized by high‐intensity appearance, accumulation, and growth of region around the ischemic territory in both MCAO and CVST models. Besides, 4T‐BSA nanoprobes extravasated unambiguously and reversibly in multiple areas of epilepsy model. Therefore, in vivo longitudinal NIR‐IIa imaging can afford valuable insight into the pathological activities of brain associated with BBB damages.

## 3 Conclusion

In conclusion, highly crystalline organic 4T‐BSA nanoprobes that emit intense fluorescence in a NIR‐IIa window can be applied for high‐fidelity dynamic in vivo monitoring of cerebral collateral perfusion and BBB evolution in different neurologic pathophysiology models. The developed NIR‐IIa imaging provides high spatial and temporal resolution to accurately investigate the biologically critical parameters (e.g., diminutive vascular structure, arterial inflow, venous outflow, and tissue perfusion) for supporting the brain function, leading to a booming of the library of in vivo imaging modalities. Serving as an accurate in vivo diagnostic technique to monitor the BBB evolution, 4T‐BSA nanoprobes bring broad opportunities to address major unmet medical needs across timely, protective, and restorative interventions for neurological diseases.

## 4 Experimental Section

### Synthesis

For all synthesis details, please see the Supporting Information.

### Animal Handling

All vertebrate animal experiments were performed under the approval of Stanford University's Administrative Panel on Laboratory Animal Care. Eight‐week‐old female C57BL/6 mice were obtained from Charles River for all imaging studies and housed at the Research Animal Facility of Stanford under the approved animal protocols. Before vessel or BBB imaging, all mice were anaesthetized in a rodent anesthesia machine with 2 L min^−1^ O_2_ gas mixed with 3% isoflurane. Tail vein injection of contrast agents was carried with a catheter and synchronized with a camera that started continuous image acquisition simultaneously. The injected dose was a 150–200 µL bolus in a 1×PBS solution at specified concentrations. During the time course of imaging, the mouse was kept anaesthetized by a nose cone delivering 2 L min^−1^ O_2_ gas mixed with 3% isoflurane. Mice were randomly selected from cages for all experiments. No blinding was performed. All groups within study contained n = 3 mice.

### Surgical Procedures of MCAO, CVST, and Epilepsy Animal Models

Middle cerebral artery occlusion (MCAO) was induced using the silicone‐tipped intraluminal thread occlusion method, using 4‐0 monofilament occluding threads with 3.5 mm length × 0.35 mm diameter silicone tips, as previously referenced.^[^ ^24^ ^]^ Briefly, an 11 mm silicone‐coated nylon thread was introduced into the left common carotid artery of an anesthetized mouse and directed into the internal carotid artery until it obstructed blood flow to the middle cerebral artery (MCA). After either a 60‐minute ischemia, animals were briefly re‐anesthetized, the filament was withdrawn, and wounds were sutured.

Cerebral venous sinus thrombosis (CVST) was induced as previously referenced with a slight modification.^[^ ^25^ ^]^ To induce venous sinus thrombosis, a 1.0 × 2.5 mm^2^ shallow cranial window was made to expose the middle‐posterior of superior sagittal sinus. A strip of filter paper soaked with 10% ferric chloride was applied to the exposed cranial window for 5 min, whereas the sham group received filter paper soaked with 0.9% saline. Subsequently, the ferric chloride strip was removed and the field flushed with 0.9% saline. The removed bone strip was replaced, sealed with bone cement, and the skin sutured.

The pentylenetetrazol‐induced seizure mice model was set up as previously referenced.^[^ ^26^ ^]^ Chemical kindling was induced by using chemical agents, pentylenetetrazol, a GABA(A) receptor antagonist. Pentylenetetrazol (Sigma, USA) was administered at a convulsive dose of 50 mg kg^−1^ with intraperitoneal injection. The behavioral characteristics, e.g., severity, latency, and duration of seizures, were observed in individual animals. After pentylenetetrazol injection, the seizure intensity was scored for 60 min.

### NIR‐II Imaging Setup

The NIR‐II fluorescence spectra were taken using a home‐built NIR spectroscopy set‐up in the 850–1650 nm region (10‐50 mW cm^−2^ laser power density, 2000 ms exposure time, 130 nm long‐pass optical filter). 1) 785 nm laser diode (CNI Laser, China). 2) 4.5 mm focal length collimator (Thorlabs; F230SMA‐B). 3) 780 nm band‐pass with 10 nm bandwidth (Thorlabs; FB780‐10‐01′’). 4) silicon camera (Hamamatsu C8484‐03G02). 5) telescope lens (Tamron 1:3.9 75 mm). 6) 785 long‐pass filter (Semrock; BLP01‐785R‐25). 7) 200 mm NIR achromat (Thorlabs; AC508‐200‐C). 8) 75 mm NIR achromat (Thorlabs; AC508‐75‐C. 9) 910 long‐pass and 1000 nm long‐pass filter (Thorlabs; FEL‐1000‐01′’, FEL‐900‐01′’). 10) InGaAs camera (Princeton Instruments; OMA V 1.7. 11) flip‐mirror.

### NIR‐IIa Imaging of Cerebral Circulatory System

NIR‐II imaging setup was described above. Before imaging, the hairs of brain of six‐week‐old C57BL/6 mice (n = 3) were shaved by using depilatory gel. Next, mice with sham/MCAO/CVST (mouse model set‐up as described in the above paragraphs) were mounted on an imaging stage beneath the laser. Then, 150 µL of 4T‐BSA nanoprobe (35 nM) was injected through tail vein. To compare the wavelength‐dependent vascular imaging quality of brain, different emission filters were used to select the collection range for NIR‐I (850 nm long‐pass filter) and NIR‐II (1000, 1100, 1200, 1300, 1350, and 1400 nm long‐pass filters), respectively. A magnification (2.5×) was performed. Brain NIR‐II fluorescence images were obtained with an exposure time of 2000 ms.

For assessment of blood perfusion in sham and different neuropathological models (MCAO and CVST), mice of different groups (n = 3) were imaged using the NIR‐II setup, respectively. Time series of fluorescence imaging video were captured at indicated time points of post injection of 4T‐BSA nanoprobes (35 nM) for 6 s followed by analysis of the dynamic fluorescence signal variations.

To explore the dynamic collateral blood perfusion and collateral flow network in different models (MCA or SSS), dynamic imaging set‐up was the same as that used for the aforementioned NIR‐IIa fluorescence brain imaging, but with lower magnification in order to cover the entire mouse head. The entire mouse head (n = 3) was mounted on an imaging stage beneath the laser, connected with an electric heating pad to maintain the temperature of the animal. Then NIR‐IIa imaging was recorded continuously during a 40 min period after injection of 4T‐BSA nanoprobes (35 nm) intravenously into C57BL/6 mouse MCAO and CVST models, respectively. Higher magnification NIR‐IIa dynamic imaging not only can glean the details of blood hypoperfusion, but also was used to track NIR‐IIa fluorophore flow secondary to the opening of collateral channel. During the whole process of imaging, the mice were kept anesthetized by a nose cone delivering 2 L min^−1^ O_2_ gas mixed with 3% isoflurane.

### NIR‐IIa Imaging of BBB Permeability

Before the imaging, the hairs of the brain of six‐week‐old C57BL/6 mice were shaved by using depilatory gel. To investigate the time course of BBB disruption with 4T‐BSA nanoprobes in MCAO/CVST/epilepsy model mice, the entire mouse head (n = 3) was mounted on an imaging stage beneath the laser, connected with an electric heating pad to maintain the temperature of the animal. Next, real‐time systematic recording with NIR‐IIa imaging was performed immediately after injection of 4T‐BSA nanoprobes (35 nm) in a NIR‐IIa window (1000 ms, 1350 long‐pass filter) at between 2 and 72 h after reperfusion (MCAO model), at every 5 min during the next 45 min (CVST model) and 1 h after pentylenetetrazol injection (epilepsy model), respectively. Imaging set‐up was the same as that used for the aforementioned NIR‐IIa fluorescence brain imaging.

### Ex Vivo Microscopic NIR‐IIa Imaging of BBB Permeability

Ex vivo NIR‐IIa imaging of BBB permeability was further performed at 72 h post‐injection of 4T‐BSA nanoprobes (35 nM) through tail vein in MCAO mice (n = 3). The mice were then perfused through the left ventricle at 15 mL min^−1^ for 1 min with ice‐cold phosphate buffer (PB, pH 7.4) and for 30 min with 4% formaldehyde in 0.1 m PBS. Next, whole mouse brain was removed from the skull, post‐fixed overnight at 4 °C and then transferred into 30% (w/v) sucrose in phosphate buffer. After equilibration in the 30% sucrose solution, the brains were embedded by optimal cutting temperature compound (Tissue‐Tek, Sakura Finetek, USA), and then sectioned coronally in the cryostat at ‐20 °C with a sliding microtome set. Immediately, e*x vivo* microscopic NIR‐IIa imaging was taken using a home‐built NIR spectroscopy with microscope setup, which was described in the Supporting Information.

### NIR‐I Imaging with ICG‐BSA

For comparison with NIR‐IIa imaging, NIR‐I imaging was performed with ICG‐BSA (35 nM, the equivalent dosage with 4T‐BSA nanoprobes in NIR‐IIa imaging) injected intravenously using a home‐built stereotactic platform fixed on a motorized 3D translational stage with at an excitation and emission wavelength of 785 and 810 nm. Experiment procedures were performed similar to NIR‐II imaging described in the above paragraph in MCAO and CVST models to exhibit the cerebral circulatory system and BBB permeability. Optical imaging was acquired at the same condition with NIR‐II imaging after injection. The resulting NIR photoluminescence was collected using Hamamsatsu C8484‐05G02 Digital Camera (Hamamsatsu Corporation).

### MRI Imaging of BBB Disruption and Injury after Animal Models Set Up

MRI measurements were performed using a 3‐T preclinical system (MR Solutions, Guildford, Surrey, UK). To assess the resulting lesions during the follow‐up period, T_2_‐weighted fast spin echo images (TR/TE  =  4800/85 ms, 1 average, field of view  =  25 mm, matrix size  =  240 × 256, slice thickness  =  1.2 mm) were acquired. A gadolinium‐chelate (Gd‐DTPA) contrast material (Omniscan Gadodiamide; Nycomed Inc., Princeton, New Jersey, USA; 0.1 mmol kg^−1^) was injected (intravenous bolus), and then post‐contrast T_1_‐weighted imaging (TR/TE  =  720/11 ms, 4 averages, field of view  =  25 mm, matrix size  =  248 × 512, slice thickness  =  0.6 mm) was performed immediately to view areas of contrast enhancement in the brain.

### Evans Blue (EB) for BBB Leakage Detection

BBB damage was determined by assessing the extravasation of EB (Sigma, St Louis, MO, USA). Immediately after MCAO/CVST model, EB (2% wt/vol in sterile PBS) was administered (3 mL kg^−1^) through the tail vein. At the end of reperfusion, the mice were transcardially perfused with ice‐cold PBS and 4% formaldehyde in 0.1 M PBS. Then, the brain was quickly taken out and sliced into 1 mm section. BBB disruption was visualized as leakage of EB, which appeared as blue on brain sections. In pentylenetetrazol‐induced seizure model, EB (3 mL kg^−1^, 2% wt/vol in sterile PBS) was injected intravenously 30 min before the intraperitoneal injection of pentylenetetrazol (50 mg kg^−1^). 60 min after pentylenetetrazol injection, the brains were perfused transcardially as the same procedure as above.

### In Vivo Biosafety Analysis

Healthy mice (n = 3) were injected with 4T‐BSA nanoprobes (35 nM). At day 7 post‐injection, these mice were anaesthetized and the eyeballs were removed, followed by collection of blood samples for blood biochemistry test. The mice were injected with PBS as a control. Subsequently, the main organs (heart, liver, spleen, lung and kidney) were harvested and fixed using 4% paraformaldehyde. Tissue samples were then embedded in paraffin, sliced (5 µm), and stained using hematoxylin and eosin (H&E). All of the obtained biopsy samples were imaged using an optical microscope (Leica).

### Statistical Analysis

The fluorescence measurement was performed to quantitate NIR fluorescence signal intensity through the Image J 1.45× software (National Institutes of Health, Bethesda, MD). The fluorescence analysis workflow using ImageJ software involved the following steps: ROI selection, intensity measurement, data normalization, and statistical analysis. Data are given as mean ± standard deviation (s.d.). Statistical analysis was performed using a two‐tailed Student *t*‐test. Statistical significance was assigned for *P* value < 0.05.

## Conflict of Interest

The authors declare no conflict of interest.

## Author Contributions

S.C. and H.C. contributed equally to this work. Z.C. and S.C. conceived, designed, and supervised the study. S.C. performed all the experiments and wrote the manuscript. B.C. analyzed the data and edited the manuscript. H.C. helped the synthesis of probes. X.L. assisted in characterization of probes. S.H., Z.F., and K.Q helped animal imaging. K.S. and F.G. helped to establish animal models. All authors discussed the results and commented on the manuscript.

## Supporting information



Supporting Information

Supplemental Movie S1

Supplemental Movie S2

Supplemental Movie S3

## Data Availability

The data that support the findings of this study are available from the corresponding author upon reasonable request.
