# Supplementary material for: Dynamic Pathophysiological Insight into the Brain by NIR‐II Imaging
Source: Adv Sci (Weinh). 2025 Mar 5;12(16):2416390. doi: 10.1002/advs.202416390 (PMC12021043; doi:10.1002/advs.202416390)
Supplement: Supplementary file 1 — Supporting Information [file ADVS-12-2416390-s001.docx]

**Supporting Information**

**Dynamic pathophysiological insight into the brain by NIR-II imaging**

**Si Chen^1,5,†^, Hao Chen^2,5,†^, Xinxin Li^2^, Shuqing He^5^, Kangquan Shou^5^, Kun Qian^2^, Zhao Fang^2^, Feng Gu^6^, Baisong Chang^*3^, Zhen Cheng^*2,4,5^**

1. Department of Neurology, Xiangya Hospital, Central South University, Xiangya Road 88, Changsha 410008, China
2. State Key Laboratory of Drug Research, Molecular Imaging Center, Shanghai Institute of Materia Medica, Chinese Academy of Sciences, Shanghai 201203, China
3. State Key Laboratory of Advanced Technology for Materials Synthesis and Processing, Wuhan University of Technology, Wuhan 430070, China
4. Bohai rim Advanced Research Institute for Drug Discovery, Yantai 264000, China
5. Molecular Imaging Program at Stanford (MIPS), Bio‐X Program, and Department of Radiology, Canary Center at Stanford for Cancer Early Detection, Stanford University, California 94305‐5344, USA
6. Department of Neurology & Neurological Sciences, Stanford University School of Medicine, California 94305-5122, USA

† These authors contributed equally.

** Correspondence and requests for materials should be addressed to B.C. (chang@whut.edu.cn), Z.C. (zcheng@simm.ac.cn)*

**Materials and synthesis of 4T.** The completely water-soluble organic NIR-II dye, CH1055 (MW 0.97 kDa), was synthesized as described previously^1^. CH1055 (1 g, 1.03 mmol) was dissolved in 100 mL of dry DMSO. Then, 5 g taurine (40 mmol) and 7 mL DIPEA (5.17 g, 40 mmol) were added. After the mixture was stirred for 2 min followed by adding 5 g HBTU (13.2 mmol). The reaction solution was stirred overnight at 25 ^o^C under a nitrogen atmosphere. After the reaction finished, 100 mL of water was added and stirred for 1 h to quench excessive HBTU. Finally, Dionex Summit high-performance liquid chromatography (HPLC, Dionex Corporation, Sunnyvale, CA, USA) system was used to purify the reaction and the main parameters of HPLC were shown as follows: 340U four-channel ultraviolet-visible absorbance detector, Dionex C4, 9.4 mm × 250 mm semi-preparative column, gradient elution starting from 5% acetonitrile and ending up with 95% acetonitrile (in water with 0.1% TFA) at 42 min, 3 mL min^-1^ flow rate, 254 nm and 650 nm detection wavelength. Overall, 1.3 g CH1055-4Taurine (named 4T, yield 93%) was produced as a green solid.

**Preparation of 4T-BSA nanoprobes.** 784 mM 4T and 78.4 mM BSA were dissolved in PBS solution, respectively. Then, 0.5 mL of 784 mM 4T was added into 10 mL of 78.4 mM BSA solution to keep the molar ratio of 4T:BSA at 1:2 for optical characterization (BSA: Sigma Aldrich, Lot# SLBR6762V). Of note, overall protein concentration should be maintained as low as possible to prevent possible gelling after heating. Vortex the solution to mix evenly. The obtained dye-protein complex was added into a sealed Eppendorf, and then was place in water baths for 10 min with different temperatures (60-80 ^o^C). If not used within a few hours, store the heated dye-protein solution at 4 ^o^C for long-term storage (4T-BSA nanoprobes).

**Characterization of 4T-BSA.** The Absorbance spectra of 4T and 4T-BSA nanoprobes were taken on an ultraviolet-visible-NIR Cary 6000i spectrometer that was background corrected for each biological media such as water and BSA protein solutions. The NIR-II fluorescence emission spectrum was captured on a home-built spectroscopy set-up by exciting 4T and 4T-BSA nanoprobes with an 808 nm laser diode with a power output of 160 mW. The excitation laser was filtered with a combination of an 850 (Thorlabs)/1,000 (Thorlabs)/1,100 (Thorlabs)/1,200 (Thorlabs)/ 1,300 (Thorlabs)/1,350 (Thorlabs)/1,400 (Thorlabs) nm long-pass filters. Samples were added to either a 1mm or 1 cm path-length cuvette and the resulting emission filtered through a 910 nm long-pass filter (Thorlabs) to reject the incident excitation laser light. The emitted fluorescence was collected on a spectrometer (Acton SP2300i) coupled to a linear liquid nitrogen cooled InGaAs detector array (Princeton Instruments, OMA-V). After collecting the raw acquisition data, a correction file was applied to correct for the variable InGaAs quantum efficiency as a function of detection wavelength as well as the variable 910 nm long-pass filter extinction features across the NIR-II spectral region. All fluorescent enhancement values were derived from measurements on the wavelength-corrected spectrometer unless specifically stated otherwise.


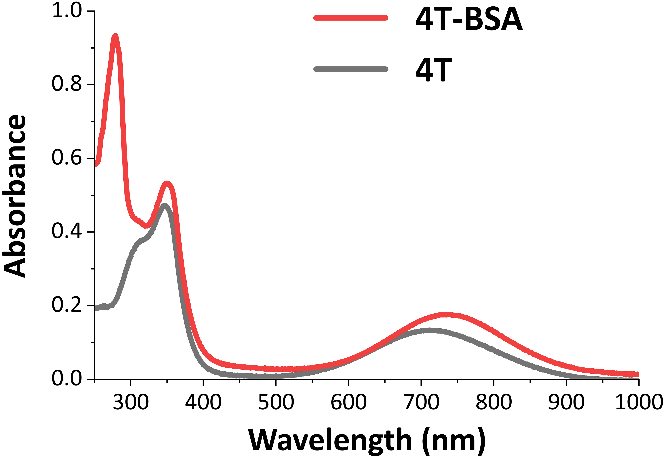


**Figure S1 |** UV-Vis spectra of 4T and 4T-BSA nanoprobe, respectively.


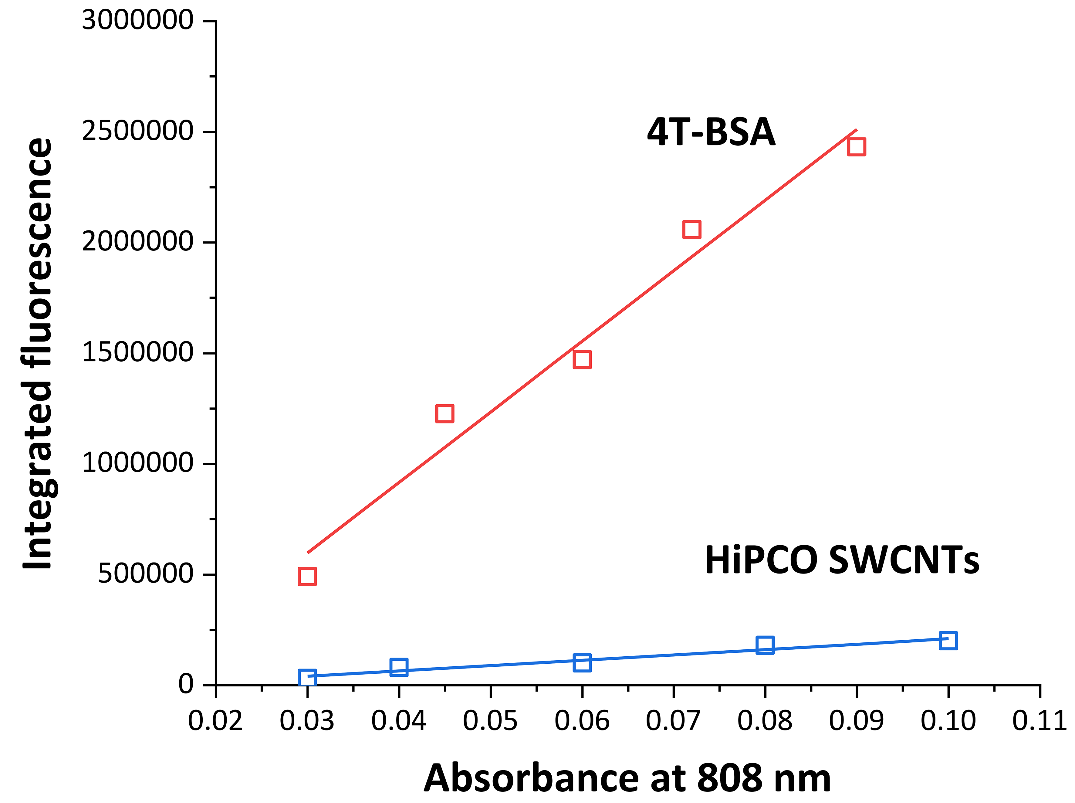


**Figure S2 | Quantum yield of 4T-BSA in PBS.** Plot of the integrated fluorescence spectrum of 4T-BSA in PBS (absorbance measured in OD). Linear fits were used to calculate quantum yield by comparing the slopes to reference HiPCO SWCNTs (QY = 0.4%).

$${QY}_{4T-BSA}= \frac{Slope of 4T-BSA}{Slope of SWCNTs} \times{QY}_{SWCNTs}$$

$=13.25 \times0.4\%=5.3\%$


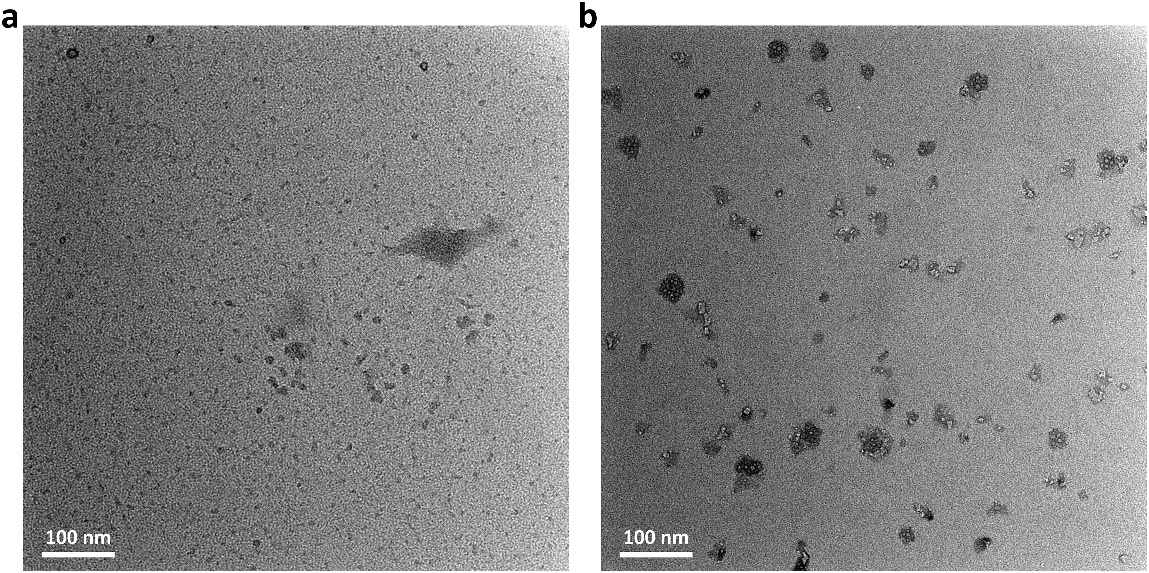


**Figure S3 |** TEM images of 4T (**a**) and BSA protein (**b**), respectively.


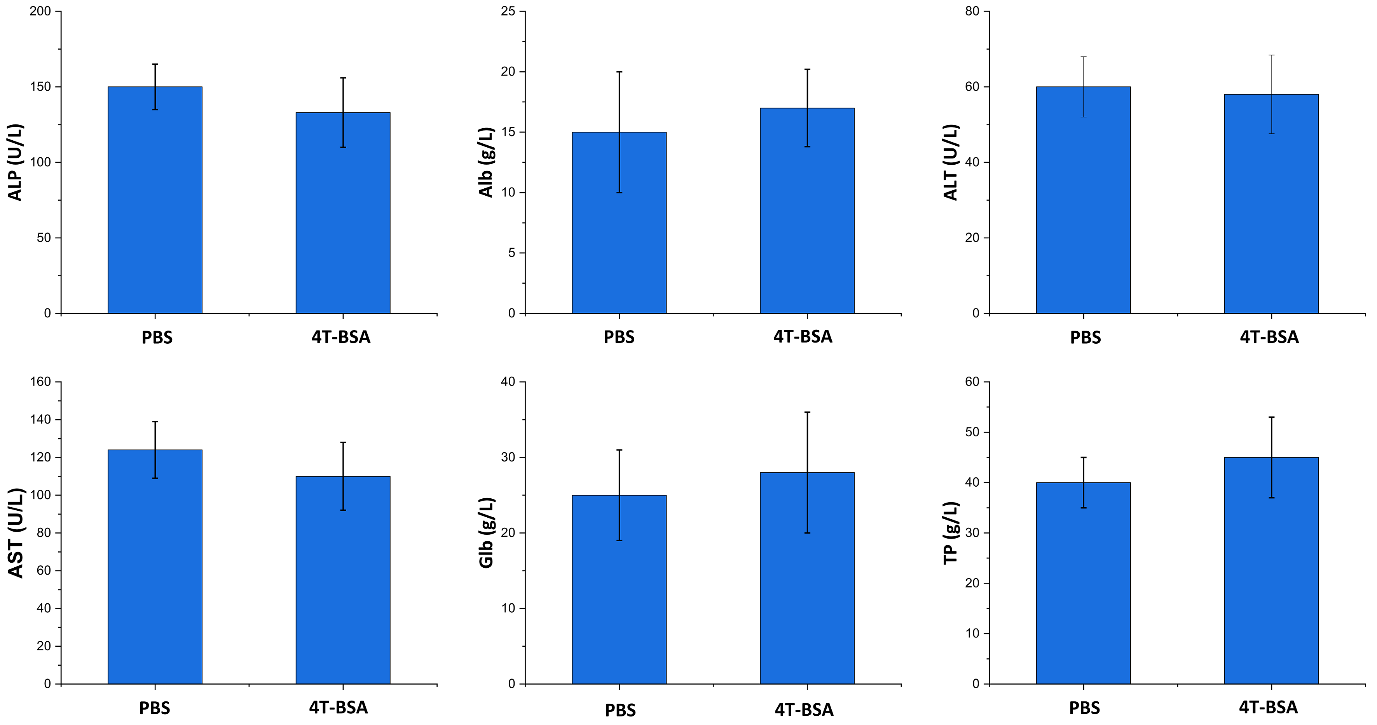


**Figure S4 | Biosafety Assessment.** Blood biochemical parameters of mice collected from those treated with 4T-BSA nanoprobes and from the control group. At 7 days post-injection, serum biochemical parameters analysis, including liver and kidney function indicators, showed no statistically significant differences between the treatment and control groups, indicating no hepatic or renal toxicity.


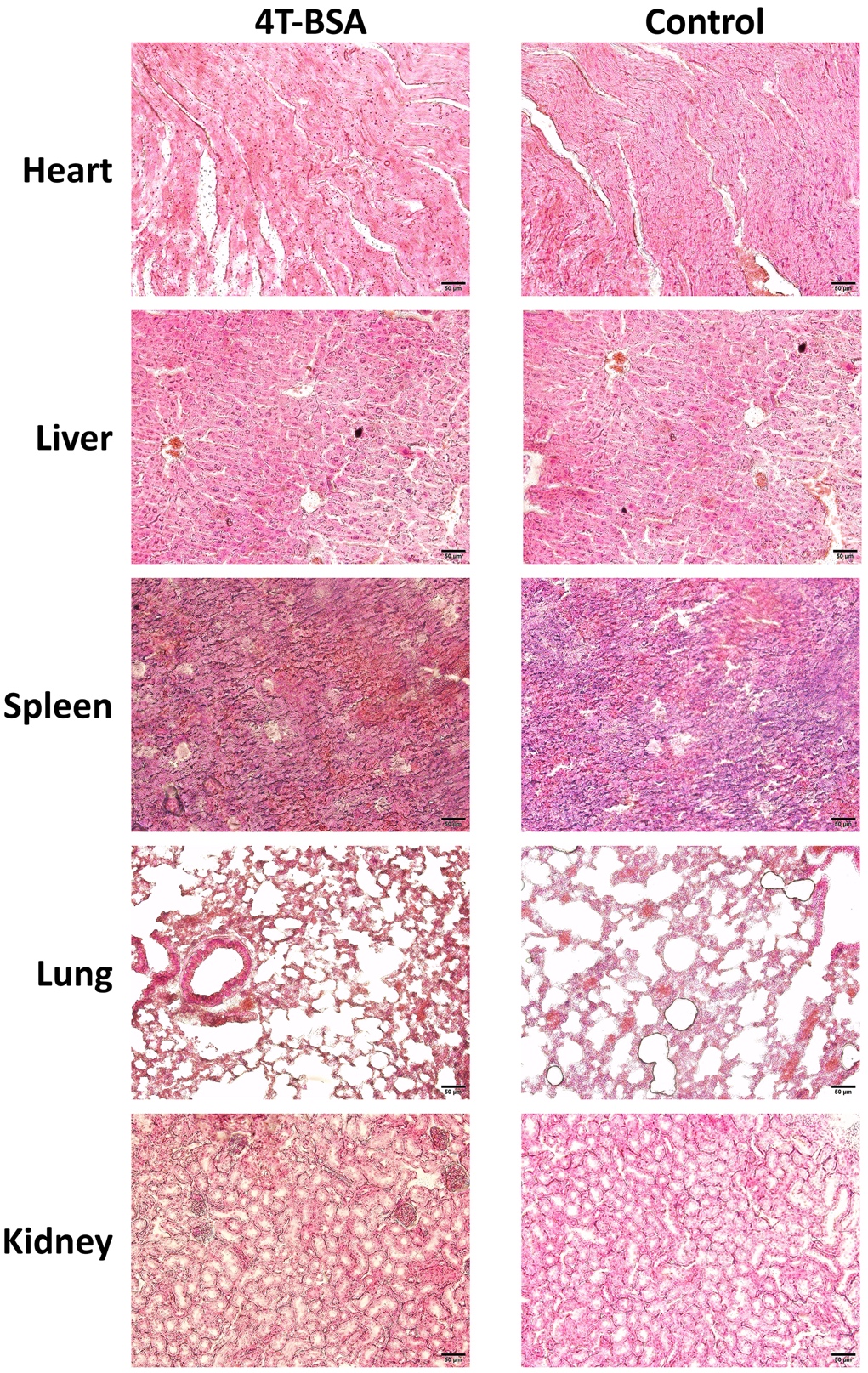


**Figure S5 | Biosafety Assessment.** H&E-stained sections of major organs collected from mice treated with 4T-BSA nanoprobes and from the control group. H&E-stained sections of major organs (heart, liver, spleen, Lung, kidney) harvested at 7 days post-injection revealed no signs of necrosis, inflammatory infiltration, or fibrosis.


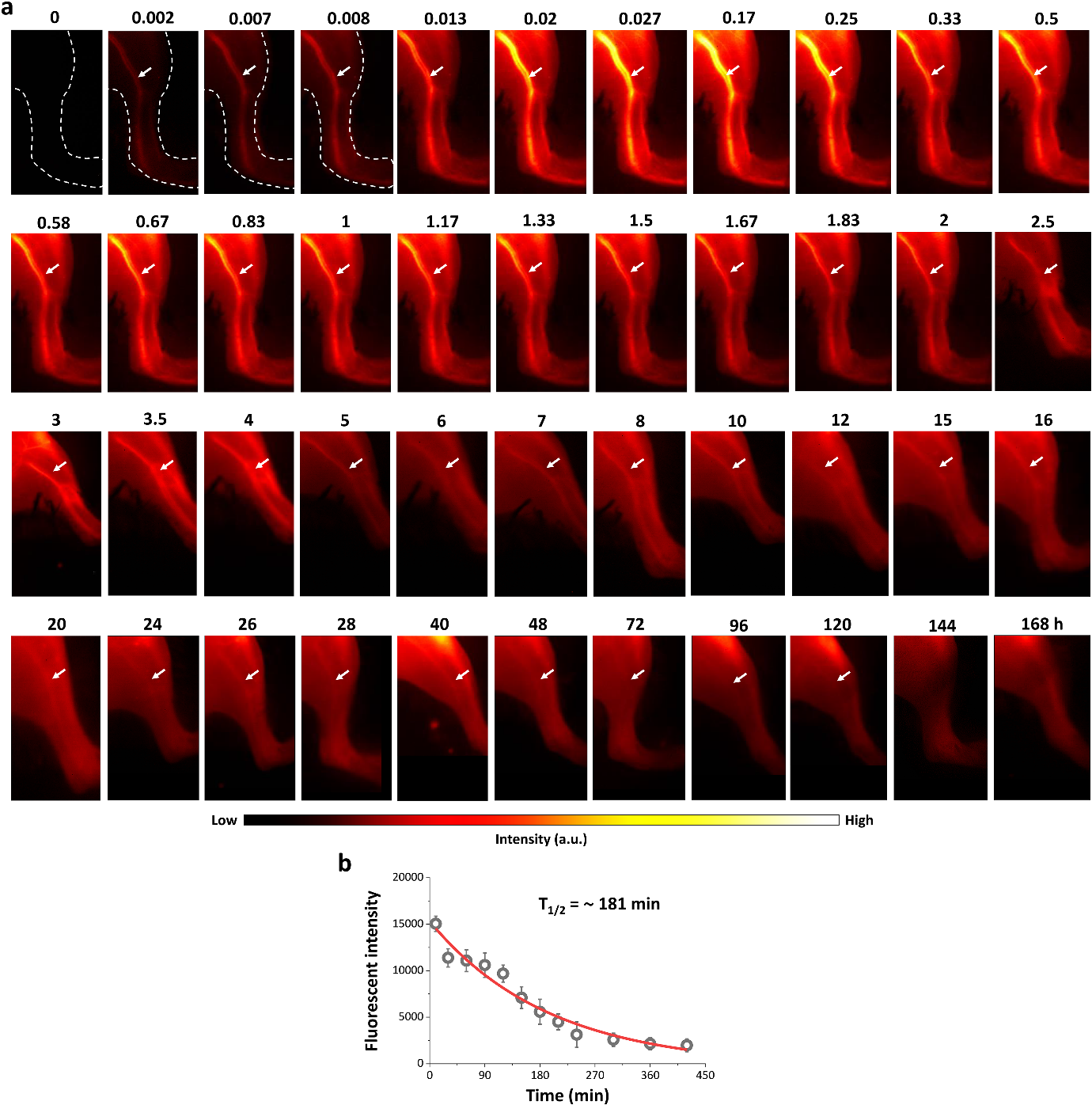


**Figure S6 | In vivo vascular ﬂuorescence images showing the hind limb (femoral artery) of a representative C57BL/6 mouse in the NIR-IIa 1350 nm window (1350 nm long-pass ﬁlter, exposure time 500 ms).** **a**, The major artery in hind limb (femoral artery) imaging showed that the long visualization of 4T-BSA nanoprobes in blood vessel during 120 h. **b**, Fluorescent intensity analysis of the femoral artery showed that the half-life of blood circulation of 4T-BSA nanoprobes was calculated to be about 181 min.


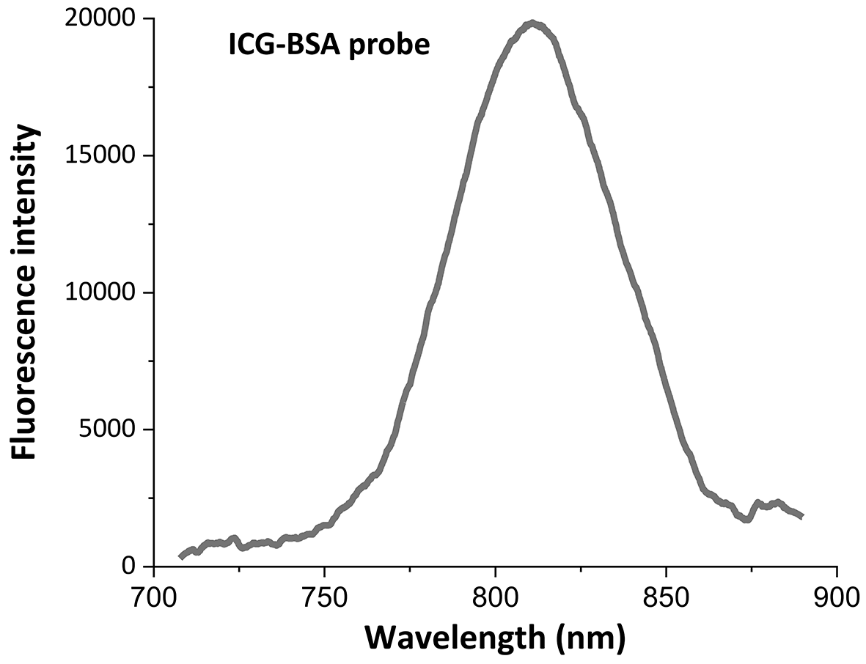


**Figure S7 |** Fluorescence emission spectra of ICG-BSA nanoprobes in PBS.


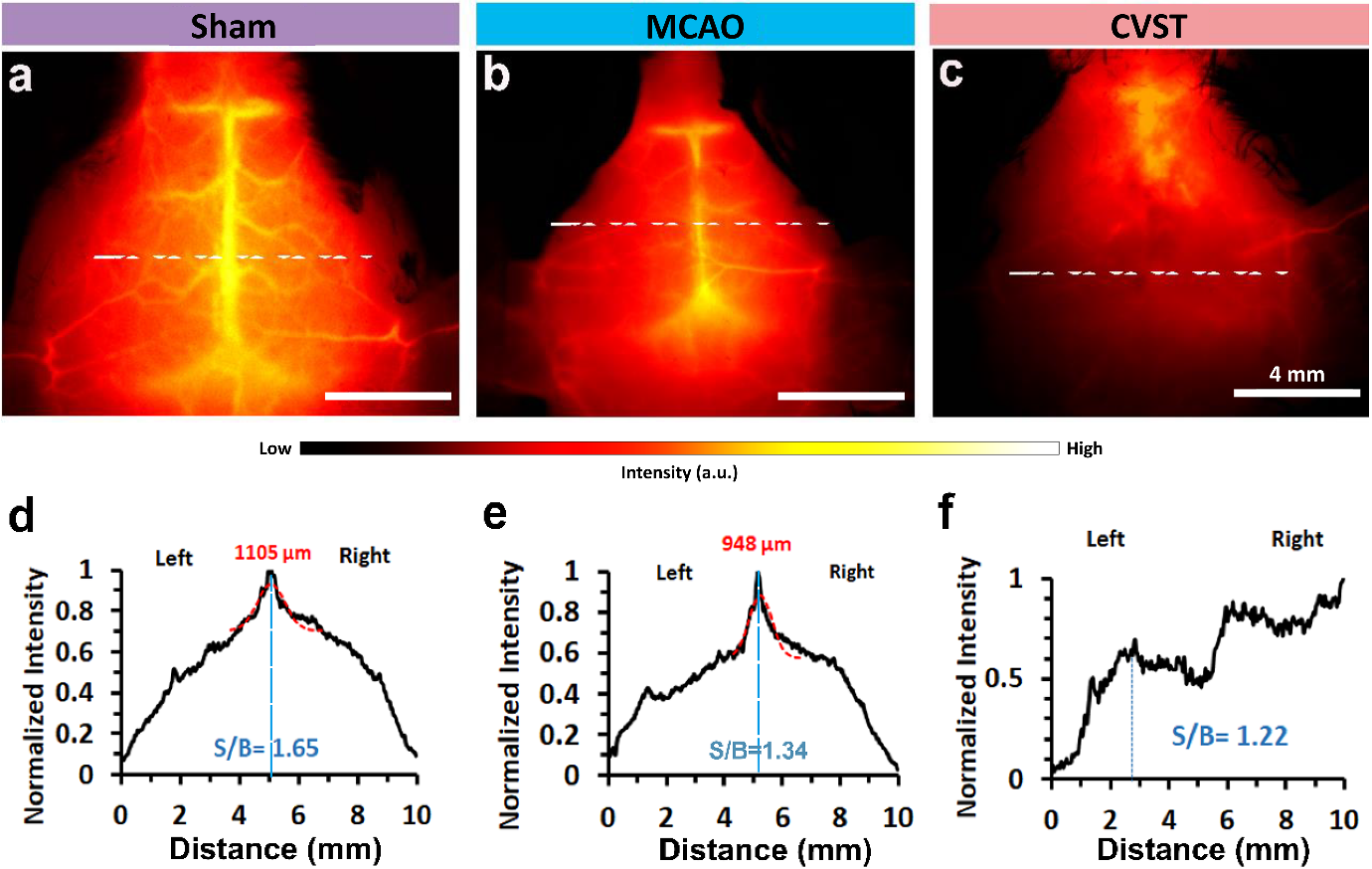


**Figure S8 | In vivo NIR-I imaging of cerebral vasculatures by ICG-BSA nanoprobe in different neurologic pathophysiological mouse models without craniotomy.** **a-c**, NIR-I fluorescence images of ICG-BSA nanoprobes acquired in sham (**a**), MCAO (**b**) and CVST (**c**) mouse models. ICG-BSA nanoprobes (35 nM) were injected intravenously into C57BL/6 mice with different neurologic pathophysiological mouse models, followed by hair shaved, then NIR-I images of mouse cerebral vasculature were obtained after injection (200 ms exposure; color bar ranges from 0 to 1200). **d-e**, The cross-sectional intensity profiles along the white-dashed lines outlined in NIR-I images (**a-c**), respectively. Quantitative studies of imaging quality were performed by fitting the recorded intensity profiles with the Gaussian function (red). The ICG-BSA nanoprobes displayed blurry vessels, and the Gaussian fit was failed in the NIR-I image on the bilateral middle cerebral artery regions in sham, MCAO and CVST models because of the poor contrast and resolution of ICG-BSA imaging.

**
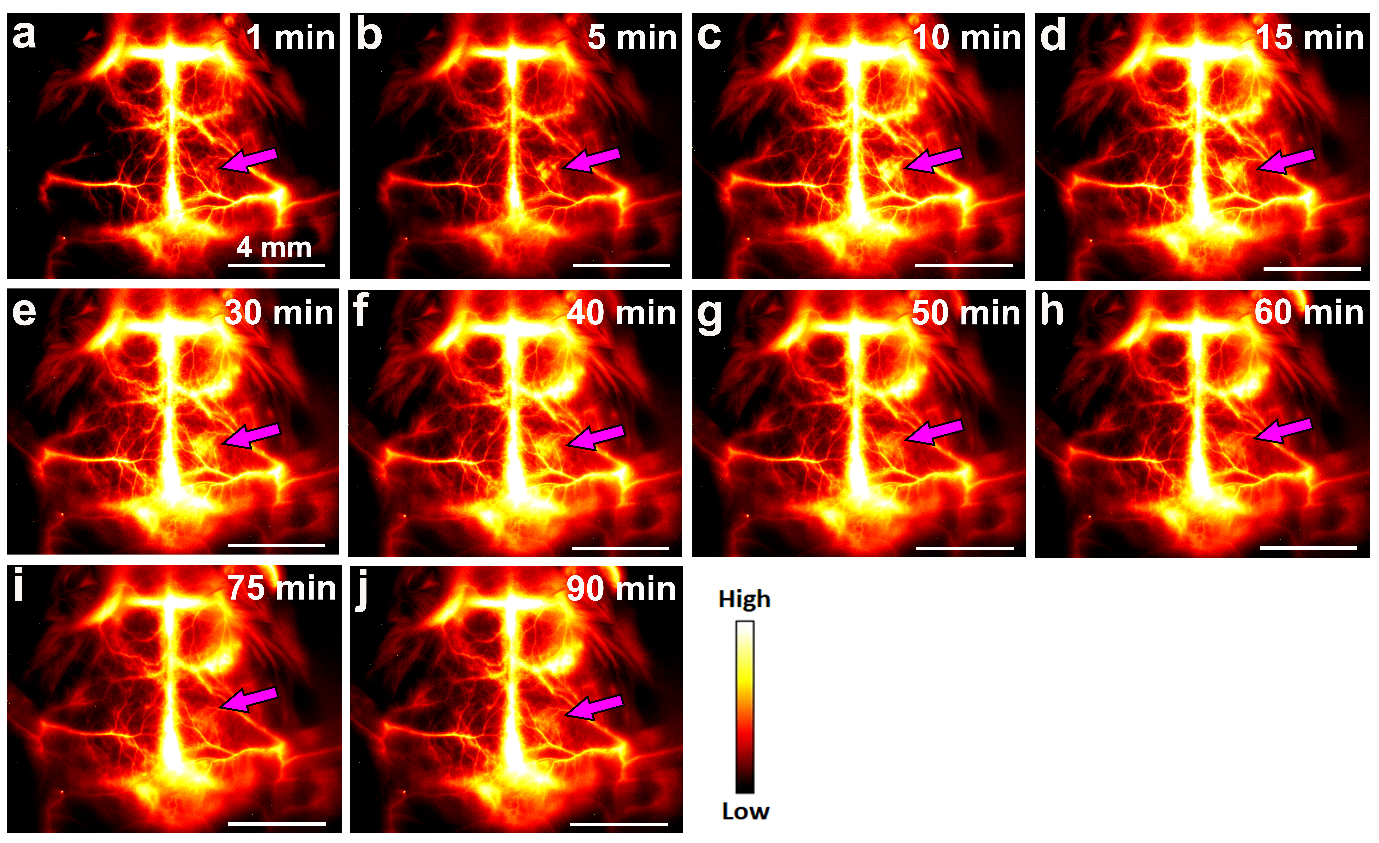
**

**Figure S9 |** In vivo dynamic NIR-IIa imaging of mouse cerebral vasculatures in the MCAO model with 4T-BSA nanoprobes. The entire mouse head was mounted on an imaging stage and recorded continuously during a 90 min period after intravenous injection of 4T-BSA nanoprobes (35 nM) into C57BL/6 MCAO mouse models (2000 ms exposure time, color bar ranges from 5000 to 35,000). The arrow indicated an angiogenesis malformation.


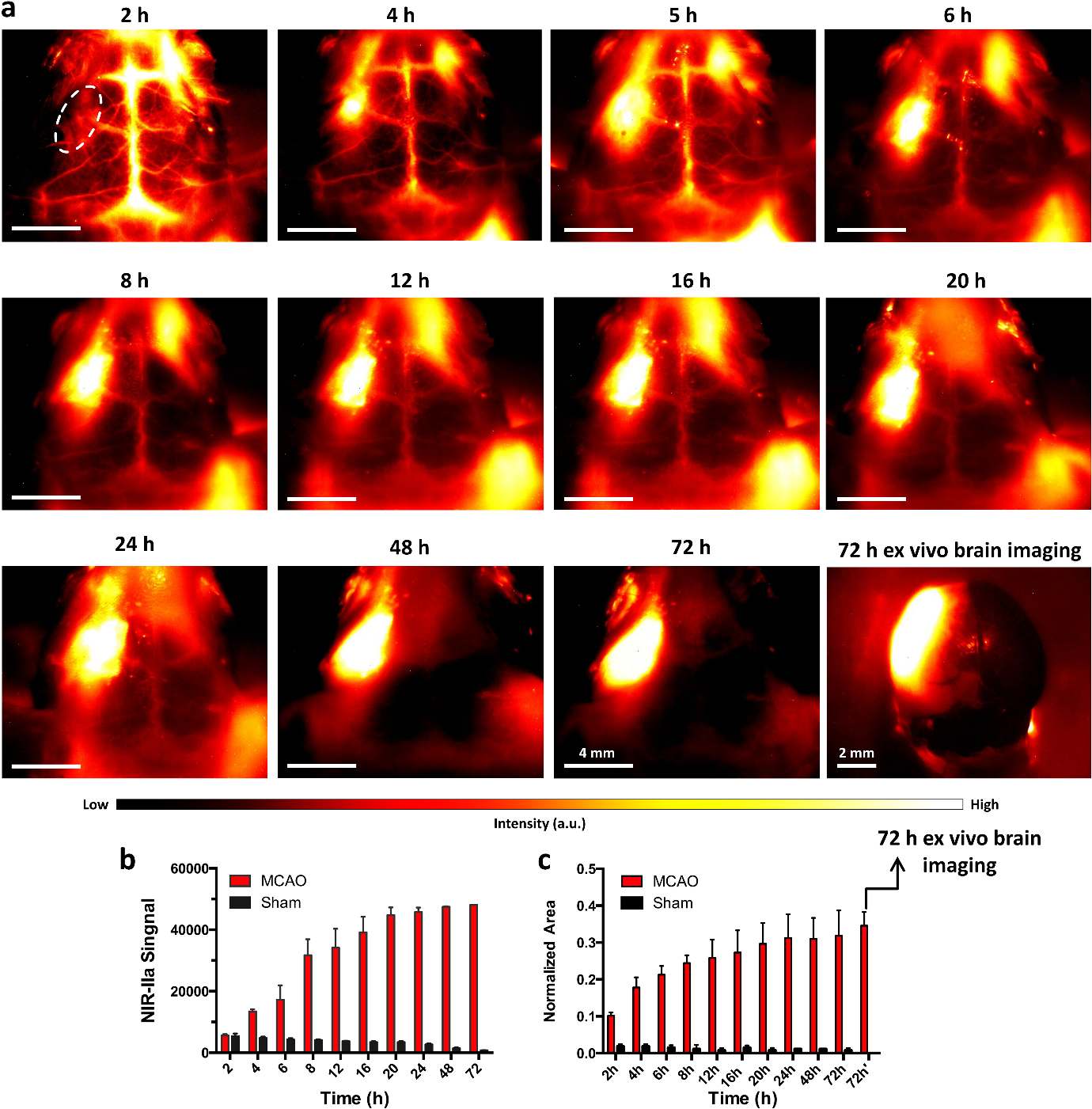


**Figure S10 | Real-time non-invasive recording of BBB permeability with NIR-IIa imaging in MCAO mouse model. a**, Non-invasive NIR-IIa brain imaging of MCAO mouse model from 2 to 72 h after reperfusion of the 4T-BSA nanoprobes. **b,c**, Time-course analysis of NIR-IIa signal intensity (**b**) and normalized signal area (**c**) from region of interest (outlined by white-dashed curve in **a**) in the left MCA territory in MCAO mouse model, respectively. To demonstrate the extravasation of 4T-BSA nanoprobes, ex vivo brain NIR-IIa imaging followed by removing the whole mouse brain from skull at 72 h after reperfusion was performed and the corresponding normalized NIR-IIa signal area was also included in **c** to evaluate the BBB permeability.


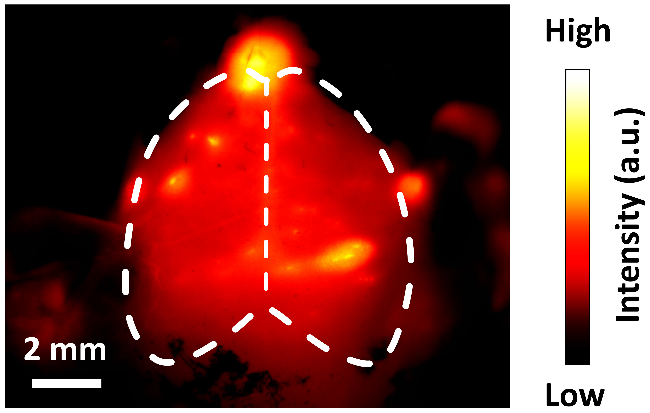


**Figure S11 | Real-time non-invasive recording of BBB permeability with NIR-I imaging in epilepsy mouse model.** In vivo NIR-I imaging (200 ms, 850 LP) of whole mouse brain through skin and skull at 2 h after ICG-BSA injection (35 nM). The fluorescence signal, deriving from BBB permeability, cannot be observed in the brain.

**Description of Additional Supporting Files**

Supporting Movie 1

Description: In vivo video-rate NIR-IIa intensity imaging of the major artery in hind limb (femoral artery) in the first 2-minute post-injection of 4T-BSA nanoprobes.

Supporting Movie 2

Description: In vivo video-rate NIR-II intensity imaging of MCAO mouse models by intravenous injection of 4T-BSA nanoprobes.

Supporting Movie 3

Description: In vivo video-rate NIR-II intensity imaging of CVST mouse models by intravenous injection of 4T-BSA nanoprobes.

**References**

1. Antaris, A.L.*, et al.* A small-molecule dye for NIR-II imaging. *Nat. Mater.* **15**, 235-242 (2016).
